# Supplementary material for: Resequencing of 672 Native Rice Accessions to Explore Genetic Diversity and Trait Associations in Vietnam
Source: Rice (N Y). 2021 Jun 10;14:52. doi: 10.1186/s12284-021-00481-0 (PMC8192651; doi:10.1186/s12284-021-00481-0)
Supplement: Supplementary file 2 — Additional file 2: Figure S1. Analysis of STRUCTURE output using the Evanno method. Evanno Plots output from Pophelper for 672 Vietnamese samples, 426 Indica samples and 211 Japonica samples. Figure S2. Mapping rate (%properly paired) for Japonica and Indica subpopulations. Figure S3. Principal coordinate analysis (PCO) of the 3635 Asian cultivated rice genomes. Plots are coloured by the subpopulations a K9_new, b K15_new. The first component represents the separation between the Indica and Japonica lines. The second components show the separation of cAus and to a lesser extent cBas while the third and fourth components represent the separation within Japonica and Indica respectively. Note for (a) we display the first 3 components and for (b) we display components 1, 2 and 4. Figure S4. Comparison between K15_3KRGP, K15_new and Vietnamese subpopulations. a Comparison between K15_3KRGP and K15_new using 3023 samples. b Comparison between K15_new and Vietnamese subpopulations using 668 samples (overlap of 56 samples from Vietnam with a). c Percentage of K15_new subpopulations from Vietnam. Arrow are shown for subpopulations which consist of > 50% of samples from Vietnam. Diagram generated using http://sankeymatic.com/. Figure S5. PCO analysis of 1605 Indica samples. Omitting the samples classified as XI-adm and Ind-adm outside Vietnam for clarity. Plot coloured by a K15_3KRGP, b K15_new including Vietnamese samples, c Five Vietnamese Indica subpopulations. The ellipses show the 95% confidence interval. X = PC1, Y=PC4, Z = PC5. Figure generated using rgl https://r-forge.r-project.org/projects/rgl/. Figure S6. PCO analysis of 982 Japonica samples. Omitting the samples classified as GJ-adm and Jap-adm outside Vietnam for clarity. Plot coloured by a K15_3KRGP, b K15_new including Vietnamese samples, c Four Vietnamese Japonica subpopulations. The ellipses show the 95% confidence interval. X = PC3, Y=PC4, Z = PC5. Figure generated using rgl https://r-forge.r-project.org/pro [file 12284_2021_481_MOESM2_ESM.pdf]

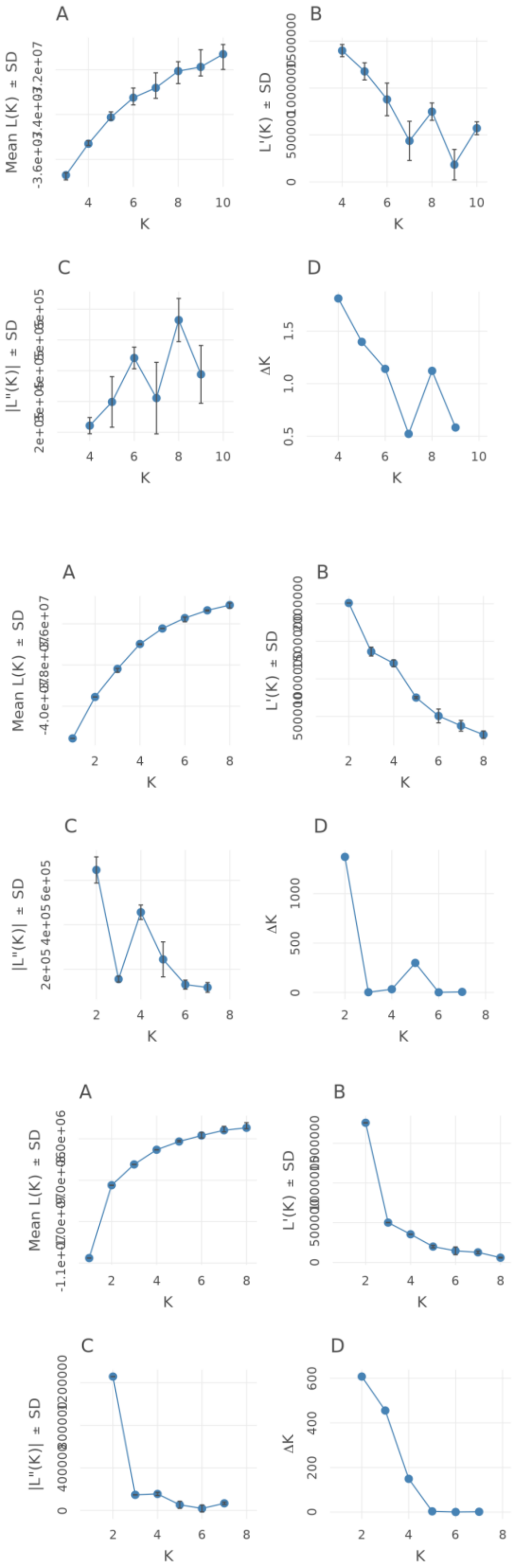

672 Vietnamese varieties

426 Indica subtypes

211 Japonica subtypes

Figure S1 Analysis of STRUCTURE output using the Evanno method.
Evanno Plots output from Pophelper for 672 Vietnamese samples, 426 Indica samples and 211 Japonica samples.

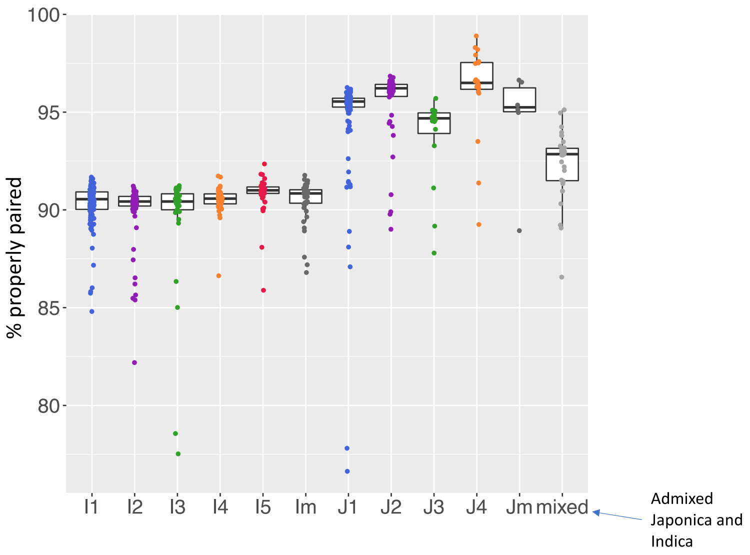

Figure S2. Mapping rate (% properly paired) for Japonica and Indica subpopulations.

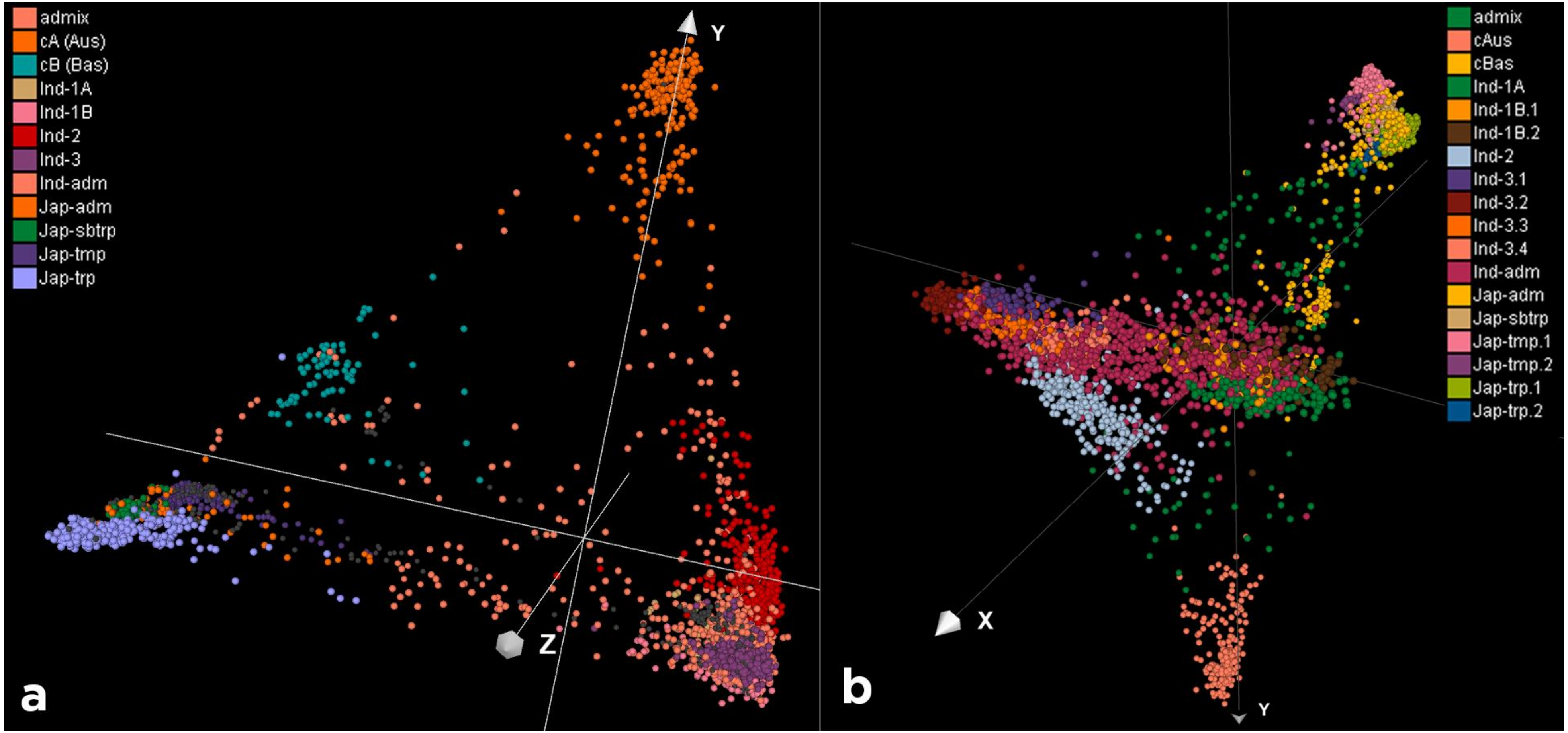

Figure S3. **Principal coordinate analysis (PCO) of the 3,635 Asian cultivated rice genomes**. Plots are coloured by the subpopulations **a** K9_new, **b** K15_new. The first component represents the separation between the Indica and Japonica lines. The second components show the separation of cAus and to a lesser extent cBas while the third and fourth components represent the separation within Japonica and Indica respectively. Note for (a) we display the first 3 components and for (b) we display components 1, 2 and 4.

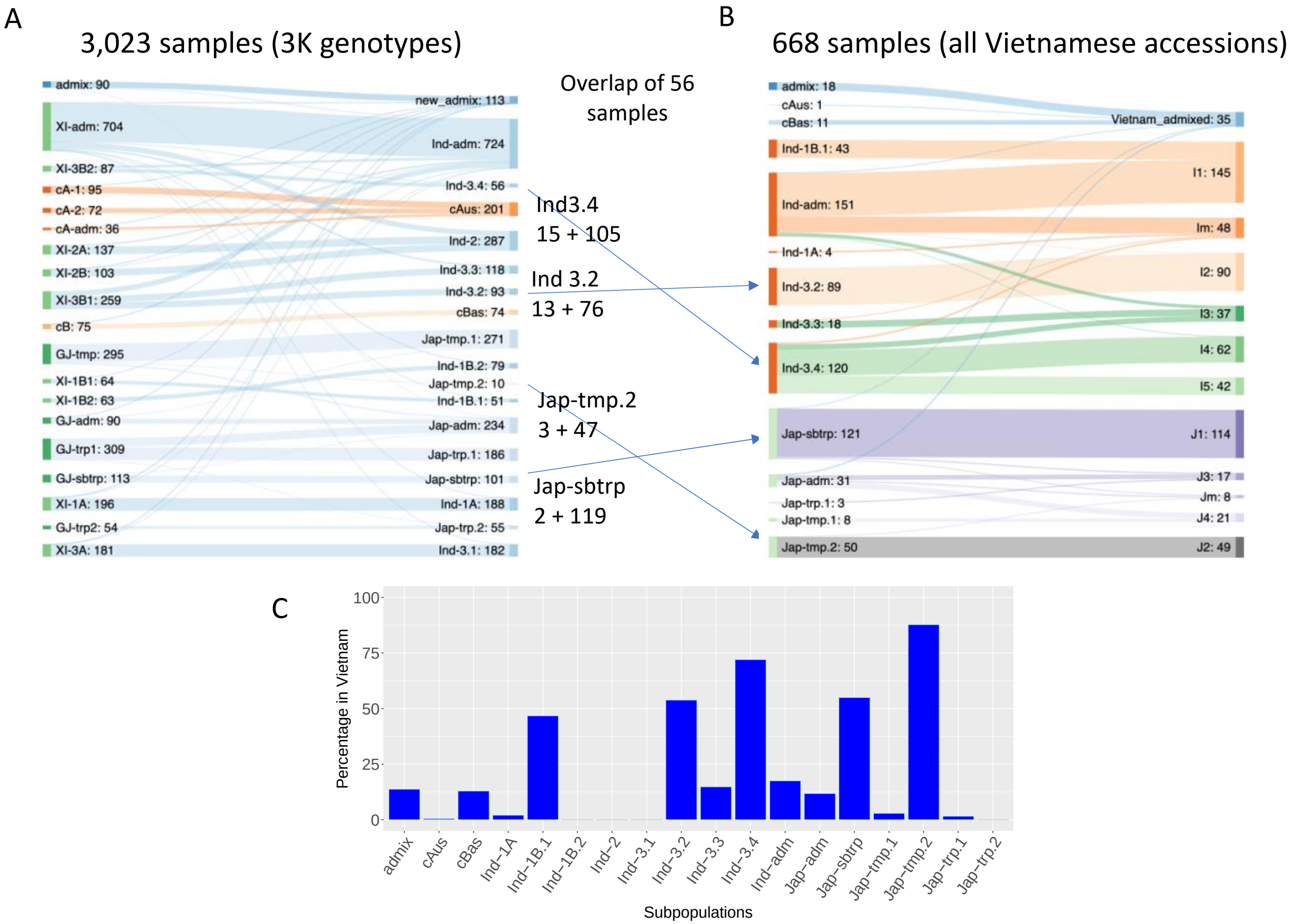

Figure S4. Comparison between K15_3KRGP, K15_new and Vietnamese subpopulations.

a Comparison between K15_3KRGP and K15_new using 3023 samples. **b** Comparison between K15_new and Vietnamese subpopulations using 668 samples (overlap of 56 samples from Vietnam with a). **c** Percentage of K15_new subpopulations from Vietnam

Arrow are shown for subpopulations which consist of > 50% of samples from Vietnam, showing the numbers of 3K RGP genotypes and newly sequenced accessions respectively

Diagram generated using http://sankeymatic.com/

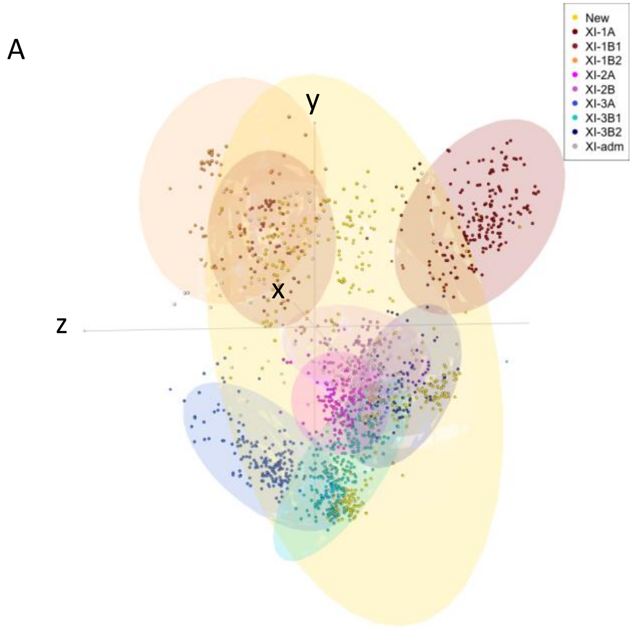

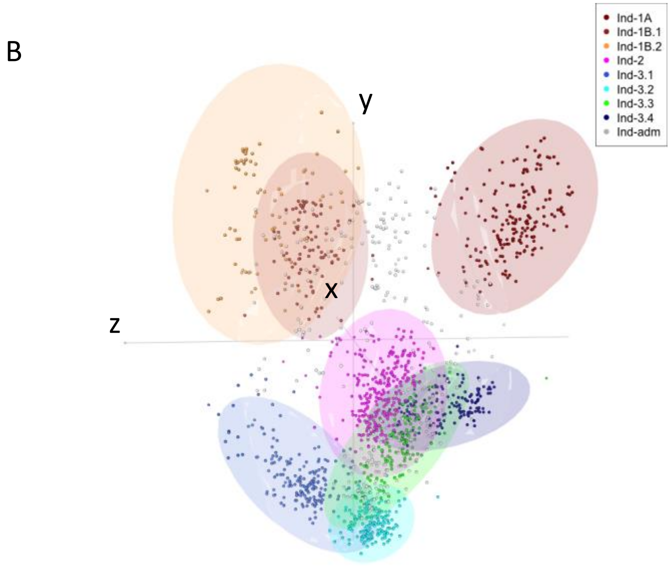

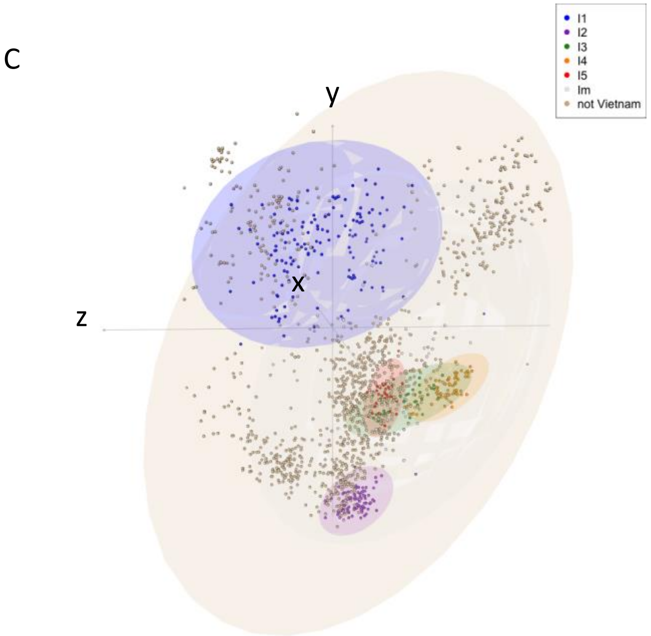

Figure S5. PCO analysis of 1605 Indica samples.
Omitting the samples classified as XI-adm and Ind-adm outside Vietnam for clarity. Plot coloured by **a** K15_3KRGP, **b** K15_new including Vietnamese samples, **c** Five Vietnamese Indica subpopulations.
The ellipses show the 95% confidence interval.
X = PC1, Y=PC4, Z=PC5.
Figure generated using rgl https://r-forge.r-project.org/projects/rgl/

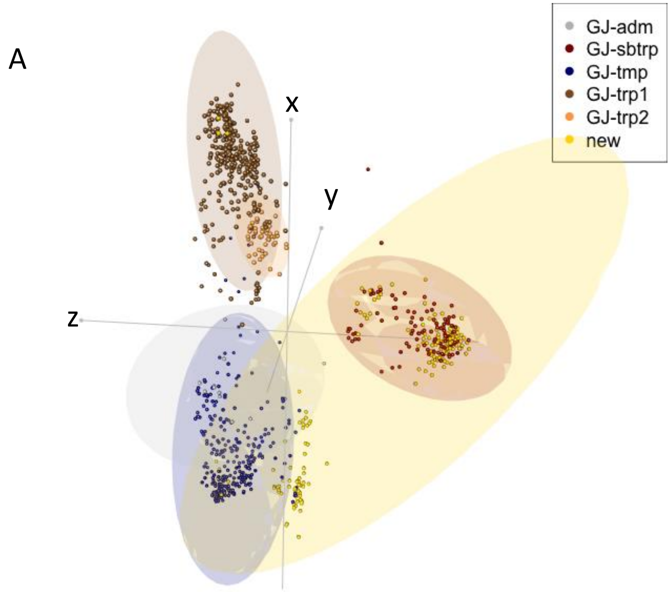

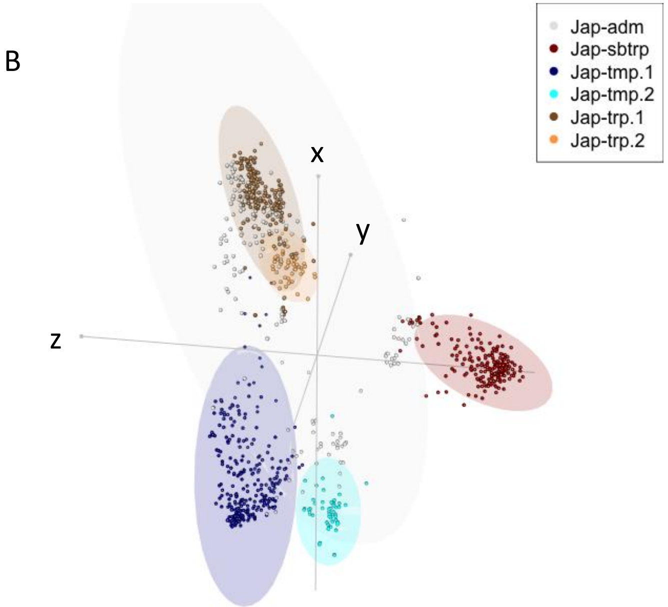

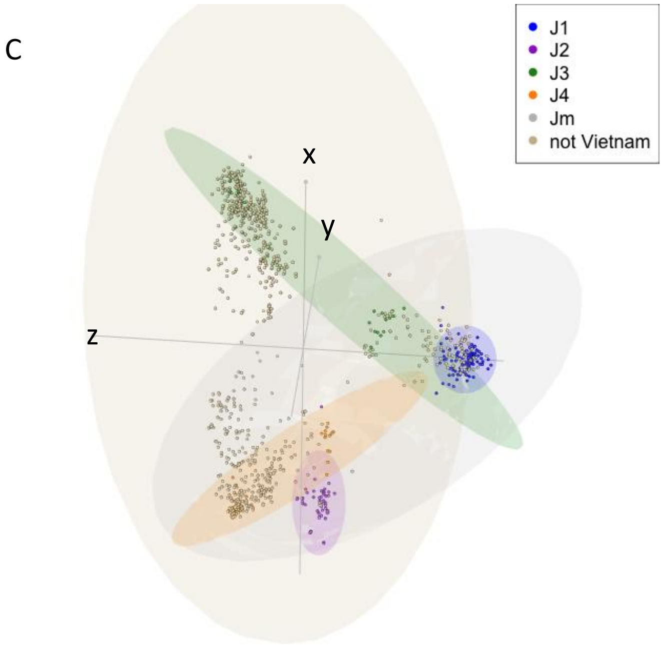

Figure S6. PCO analysis of 982 Japonica samples.
Omitting the samples classified as GJ-adm and Jap-adm outside Vietnam for clarity. Plot coloured by
a K15_3KRGP, **b** K15_new including Vietnamese samples,
c Four Vietnamese Japonica subpopulations.
The ellipses show the 95% confidence interval.
X = PC3, Y=PC4, Z=PC5.
Figure generated using rgl https://r-forge.r-project.org/projects/rgl/

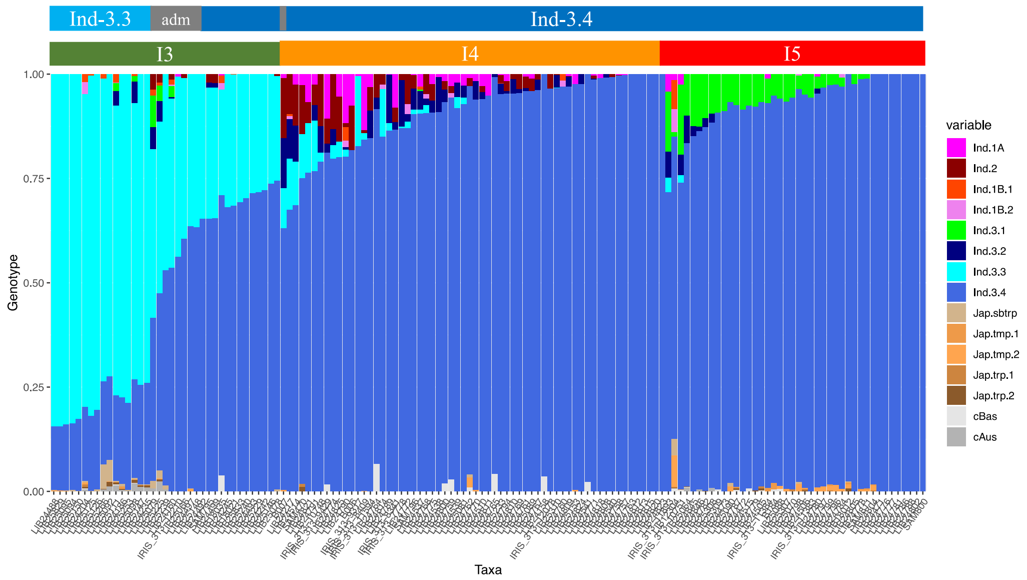

Figure S7. Admixture components of the Indica I3, I4 and I5 subpopulations.

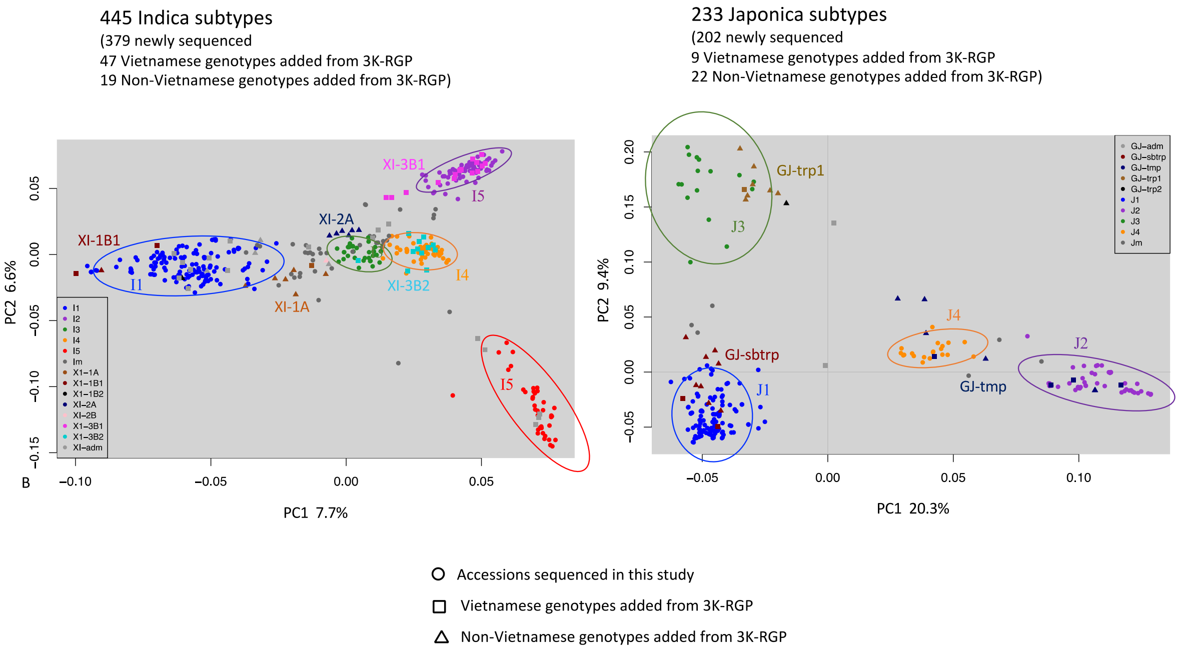

Figure S8. PCA analysis of Indica and Japonica Vietnamese subpopulations including 51 genotypes from outside Vietnam.

a PCA analysis of 445 accessions using the top two components to separate the five Indica subpopulations. The ellipses show the 95% confidence interval. **b** PCA analysis of 233 accessions using the top two components to separate the four Japonica subpopulations. The ellipses show the 95% confidence interval.

LmPb	Floret_Pubescence
LmPc	Floret_Colour
An	Awning
PnT	Panicle_Type
PnL	Panicle_Length
CmL	Culm_Length
DBI	Diameter_Internode
CmA	Culm_Angle
CmN	Culm_Number
FLA	Flag_Leaf_Angle
LA	Leaf_Angle
LBP	Leaf_Pubescence
LW	Leaf_Width
LL	Leaf_Length
PnEx	Panicle_Exsertion
CmS	Culm_Strength
HD	Heading_Date
GL_GW	GL_GW_ratio
GrW	Grain_Width
GrL	Grain_Length

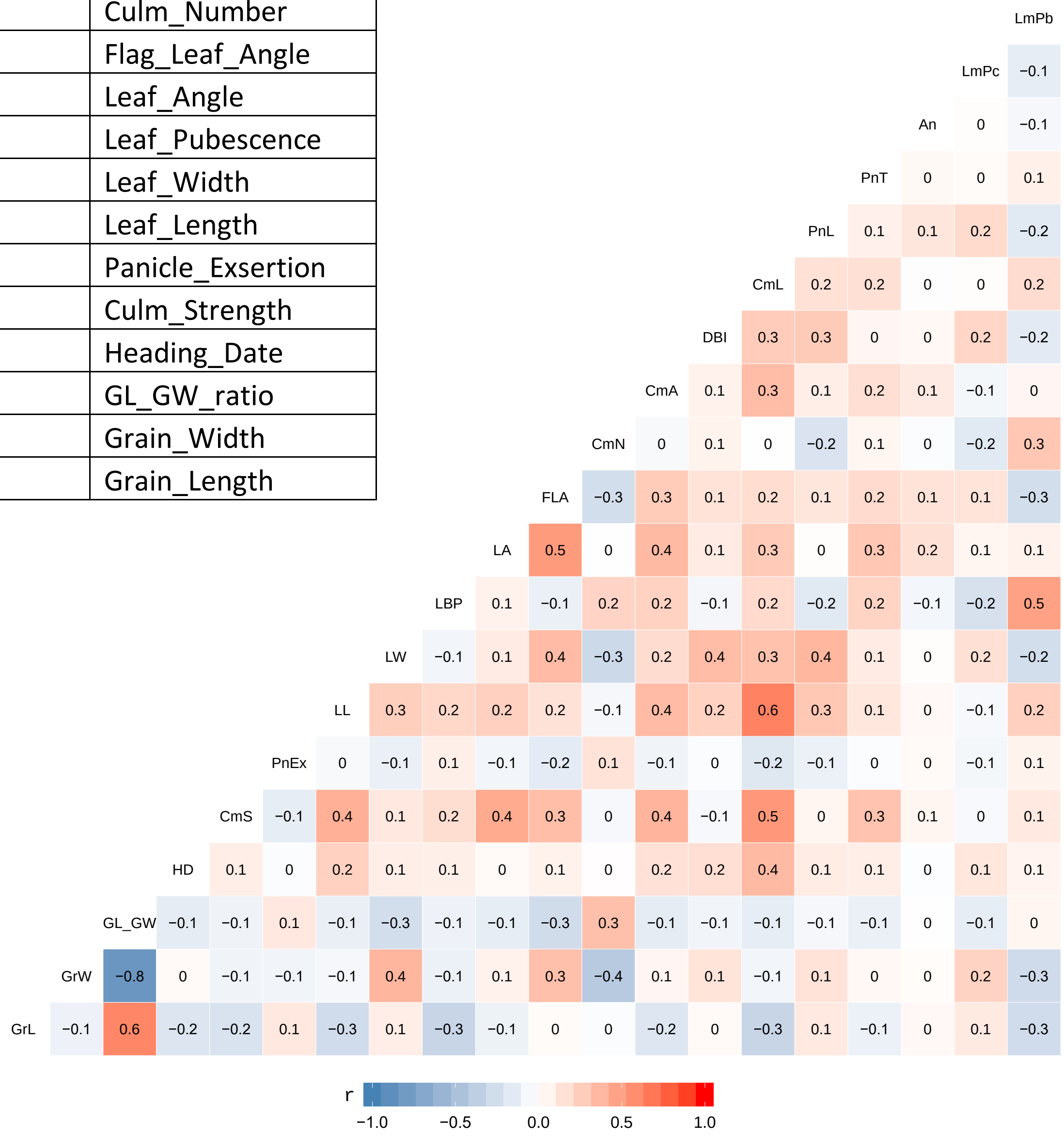

Figure S9. Correlation between the 20 phenotypes.

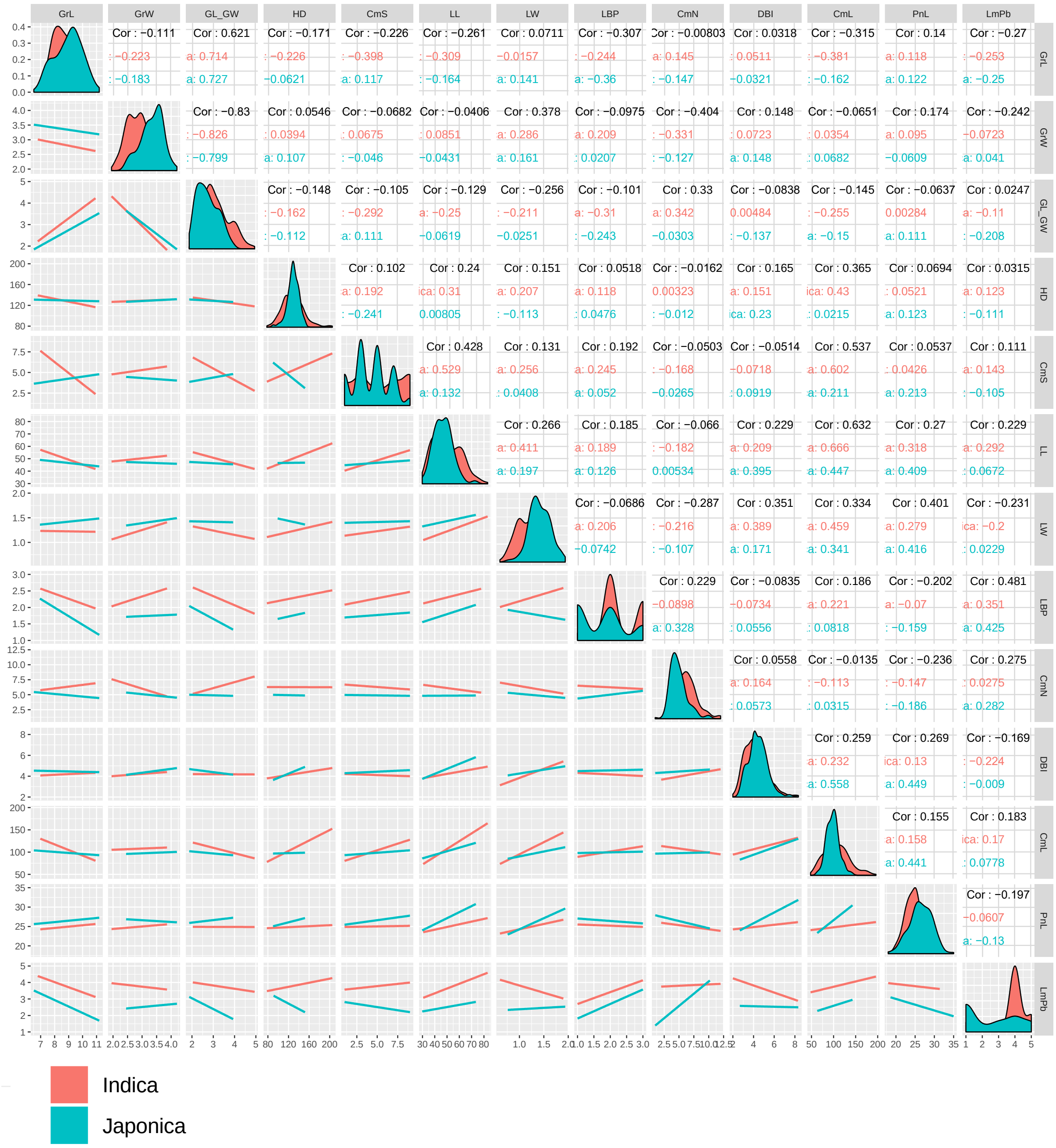

Figure S10. Correlation between Indica and Japonica for the 13 phenotypes used for GWAS
The figure was created using “ggpairs” package in R.

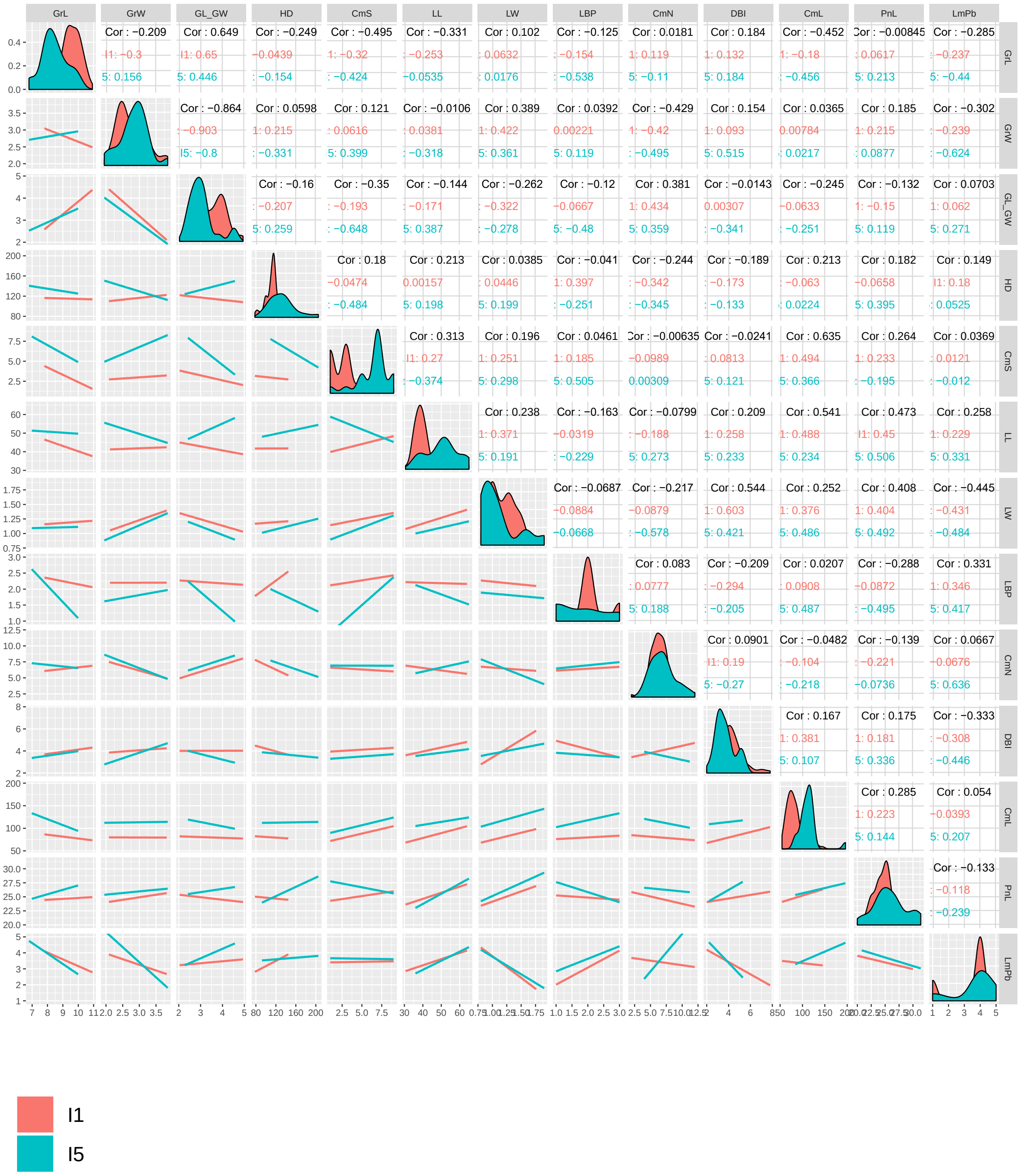

Figure S11. Correlation between Indica I1 and I5 subpopulations for the 13 phenotypes used for GWAS.

The figure was created using “ggpairs” package in R.

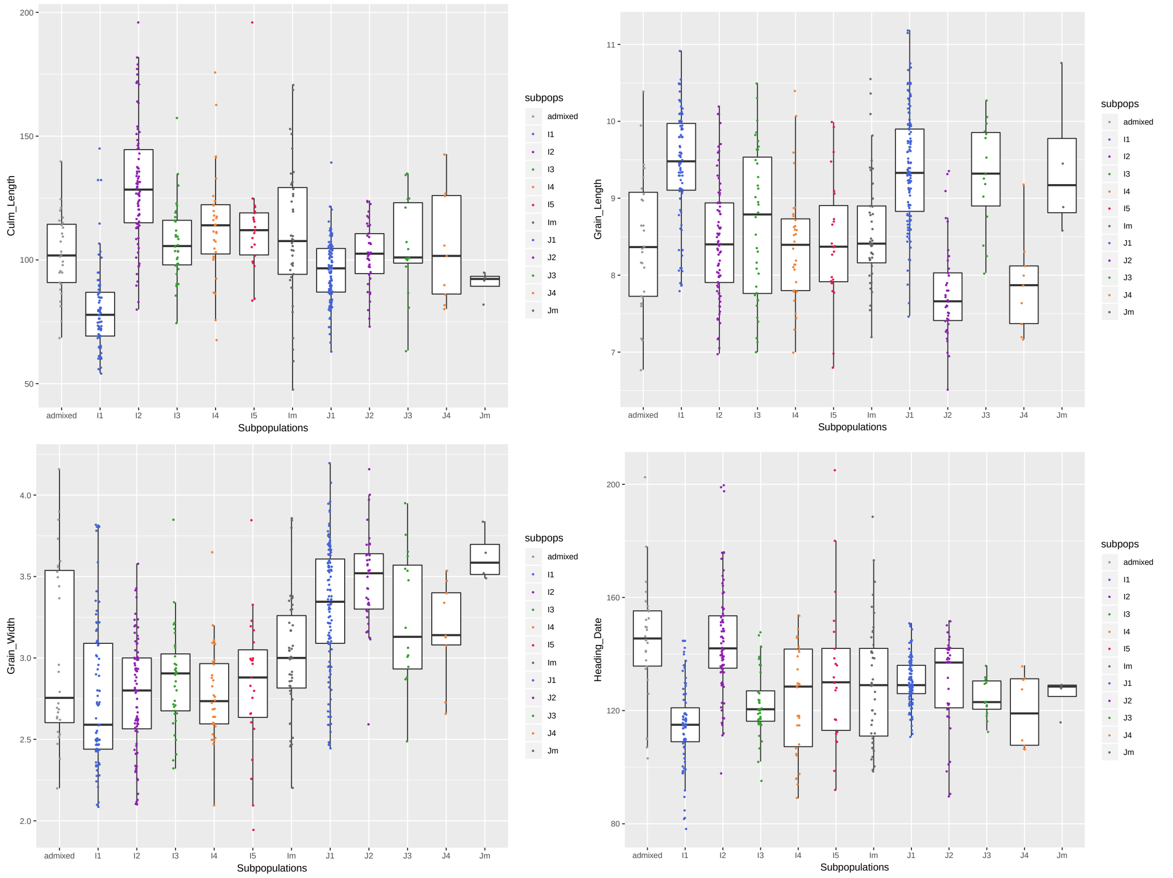

Figure S12. Boxplots showing the Phenotypic distribution per subpopulation for Culm Length, Grain Length, Grain Width and Heading Date.

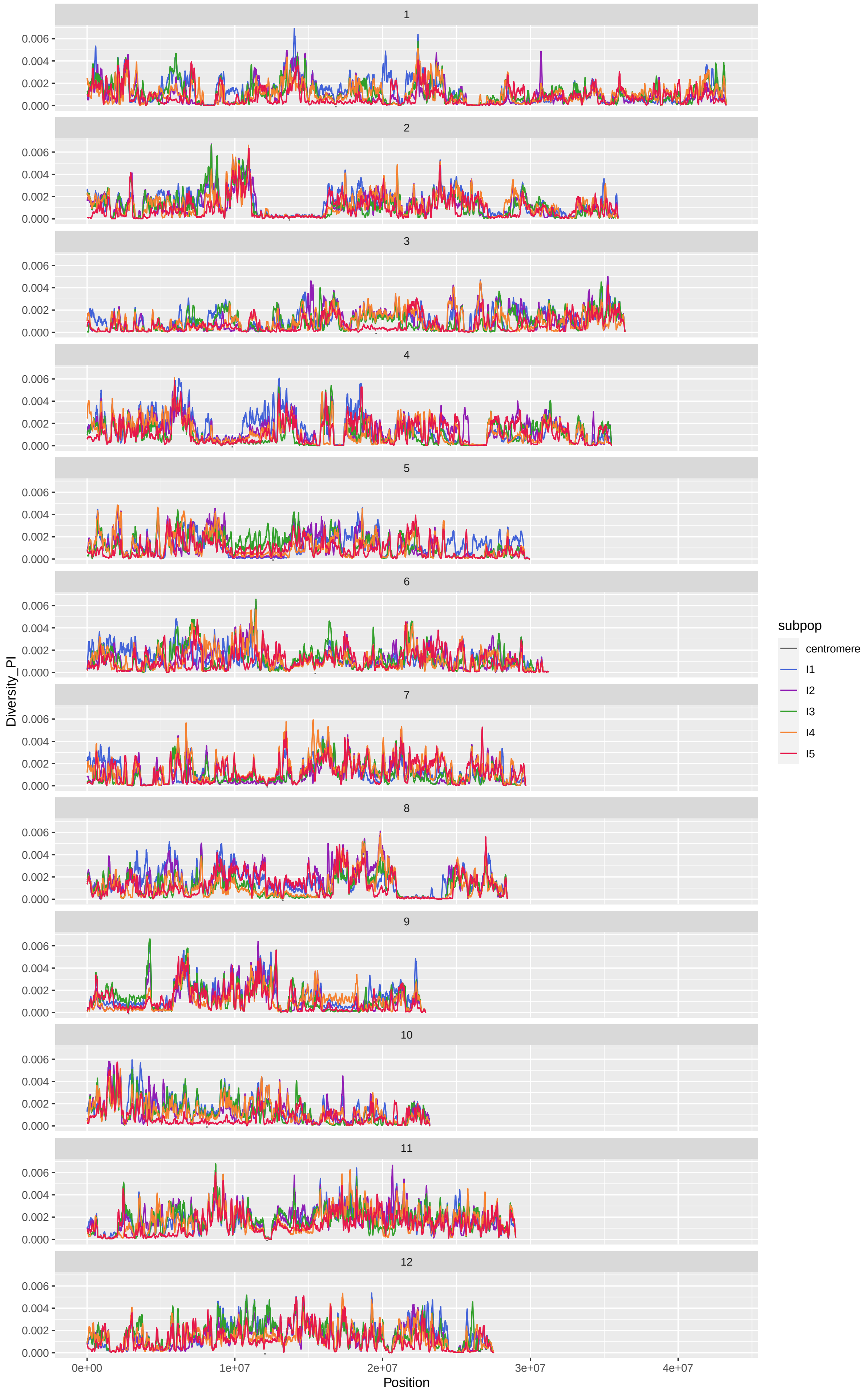

Figure S13. Indica subpopulation diversity.

Diversity (π) plotted along the 12 rice chromosomes in sliding 100kb windows.

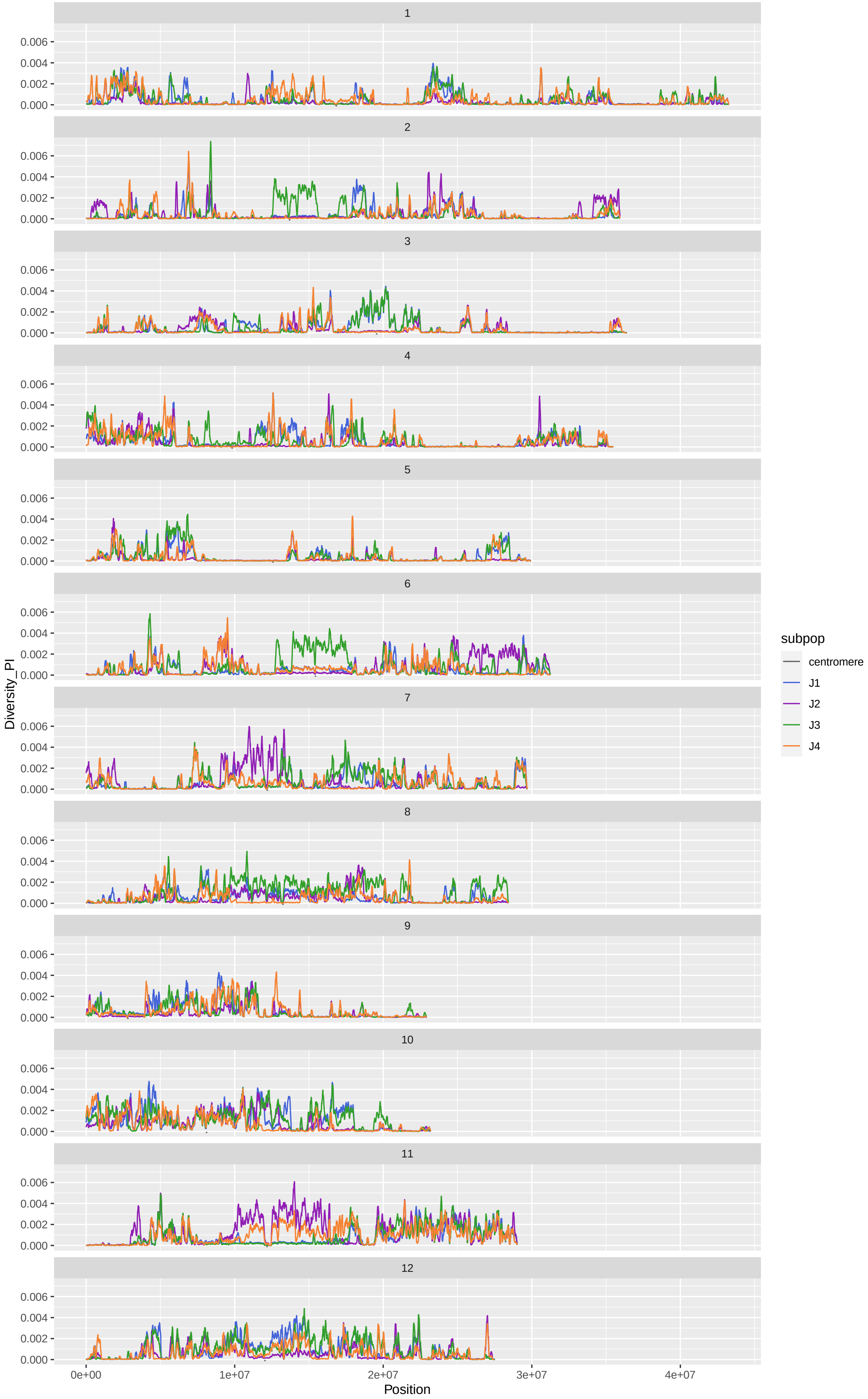

Figure S14. Japonica subpopulation diversity.
Diversity (π) plotted along the 12 rice chromosomes in sliding 100kb windows.

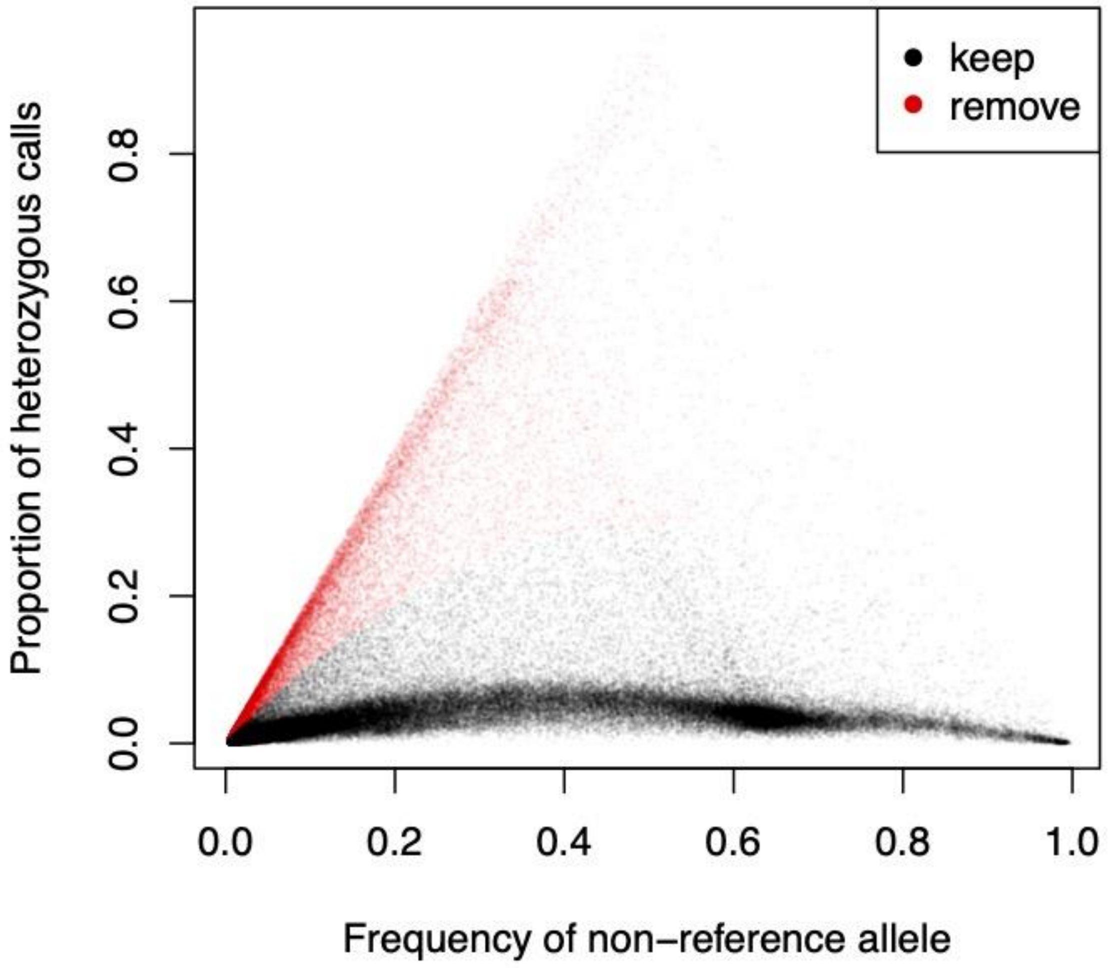

Figure S15. SNP filtering for heterozygosity

Proportion of heterozygous calls versus allele frequency. Each dot represents a SNP from a random sample of 100,000 SNPs. The points have an opacity of 5% to highlight regions of higher point density. The bulk of the SNPs lie on the Hardy-Weinberg equilibrium curve scaled by a factor of around 0.118, which implies a Wright's inbreeding coefficient of F=0.882. The SNPS have been filtered using cut off of 0.592 (5*(1-F)), the corresponding SNPs which are kept and removed are shown on the plot.

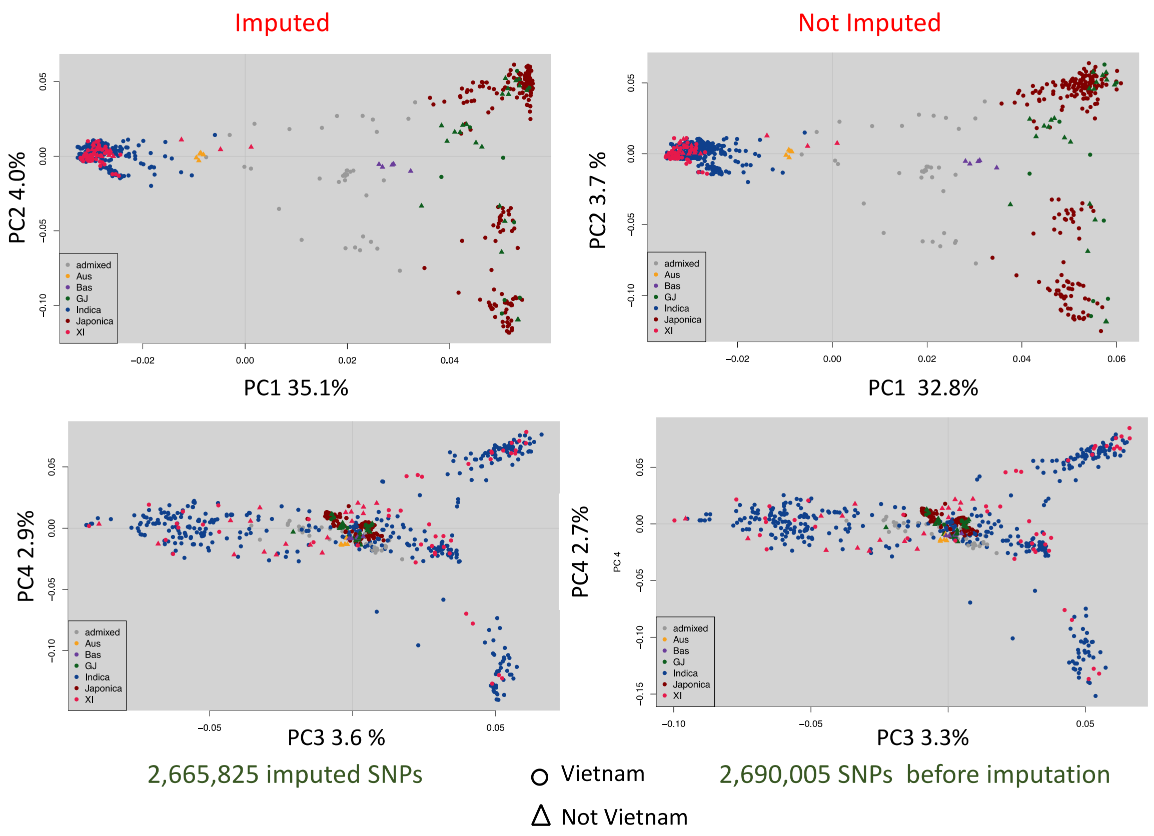

Figure S16. PCA analysis of 723 samples before and after imputation.
Comparing the 2,690,005 not imputed SNP set 3 to the 2,665,825 imputed SNP set 4
Both SNP set were filtered for 5% MAF.
Using PC1 and PC2 to separate the Japonica subpopulations.
Using PC3 and PC4 to separate the Indica subpopulations.
