# Supplementary material for: Resequencing of 672 Native Rice Accessions to Explore Genetic Diversity and Trait Associations in Vietnam
Source: Rice (N Y). 2021 Jun 10;14:52. doi: 10.1186/s12284-021-00481-0 (PMC8192651; doi:10.1186/s12284-021-00481-0)

Figure S17. GWAS Manhattan and qq plots for the full panel and Indica and Japonica subpanels for Grain Length, Grain Width, Grain length-to-width ratio, Heading Date, Culm Strength, Leaf Length and Leaf Width.

Full Panel
672 samples
501 phenotypes

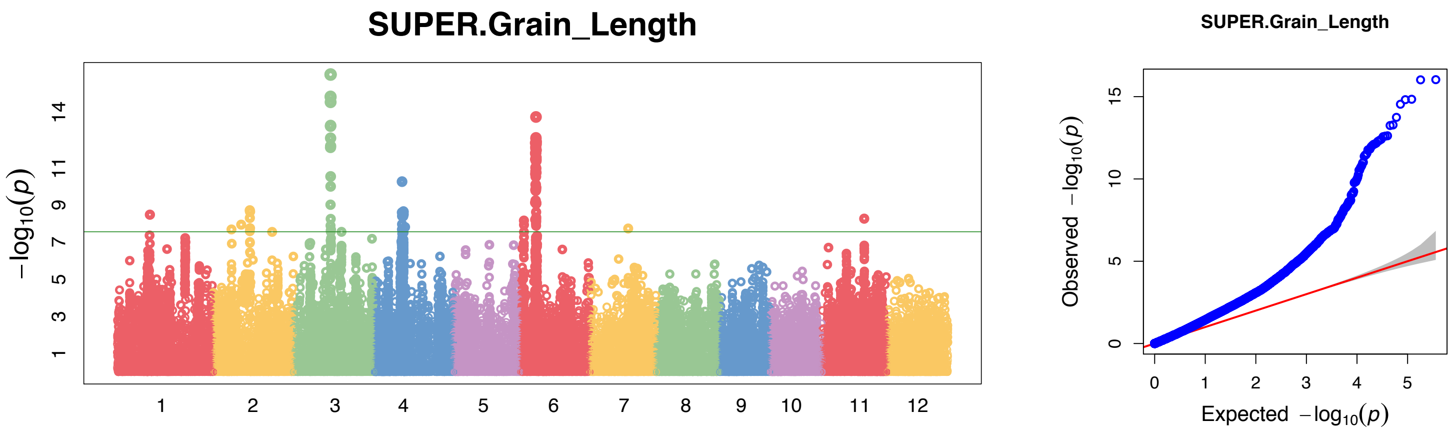

Indica Panel
426 samples
295 phenotypes

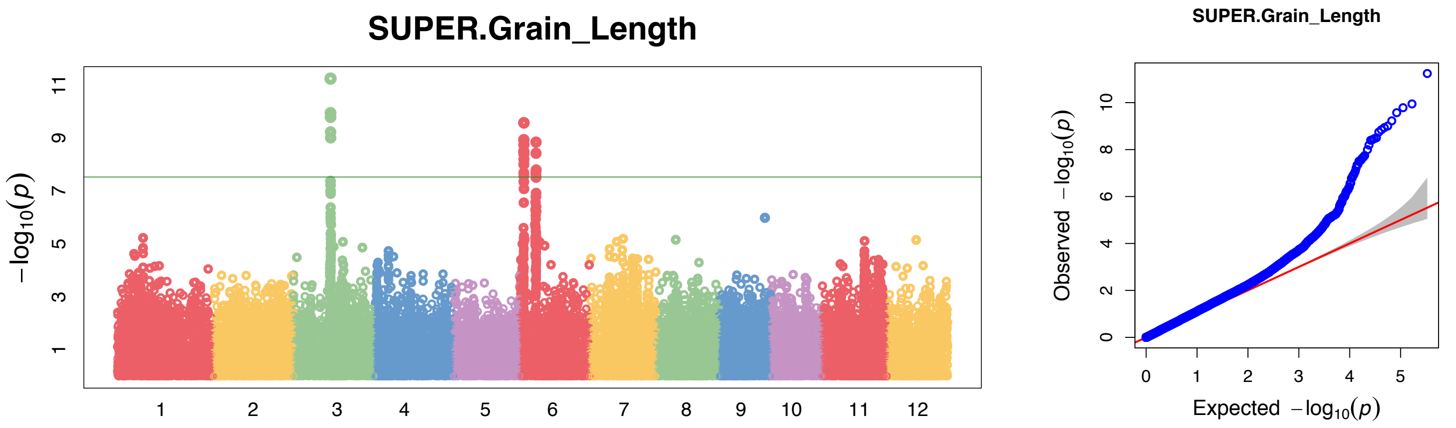

Japonica Panel
211 samples
178 phenotypes

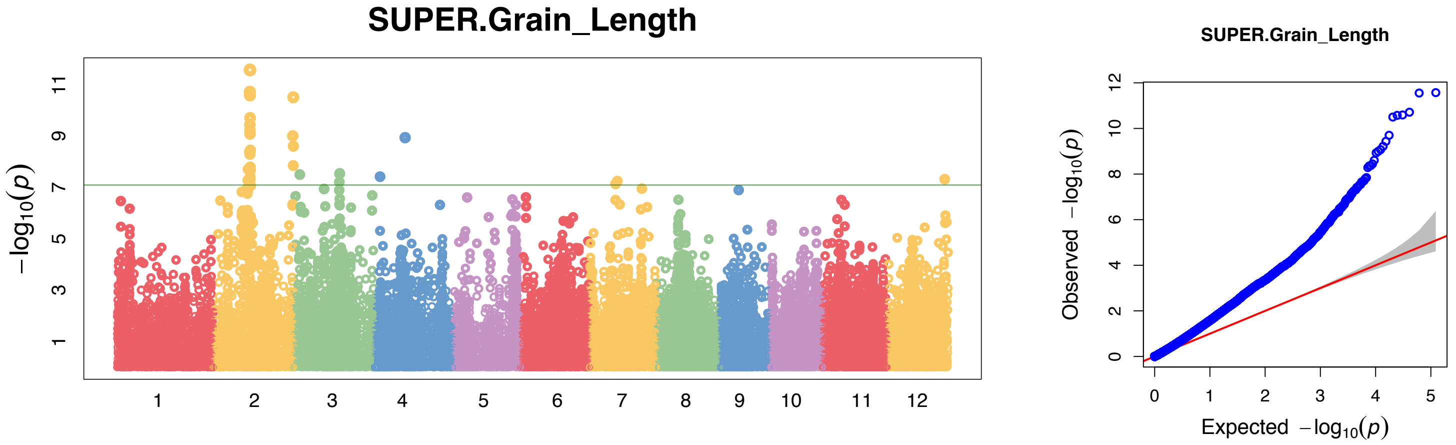

Full Panel
672 samples
503 phenotypes

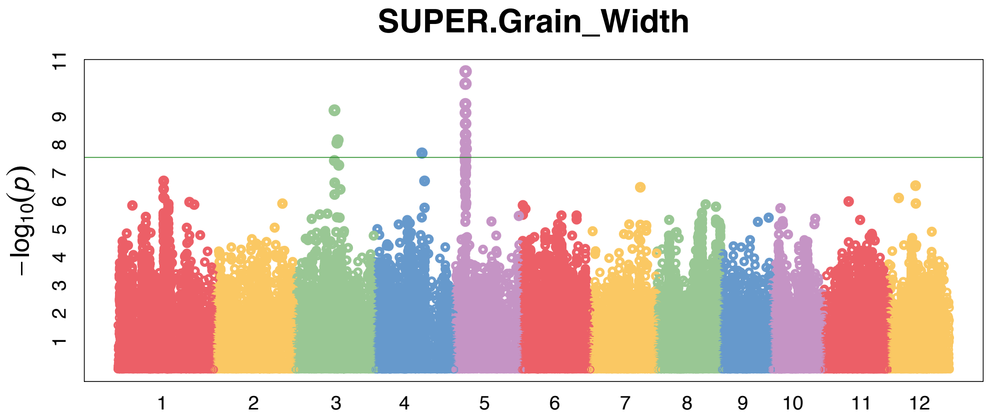

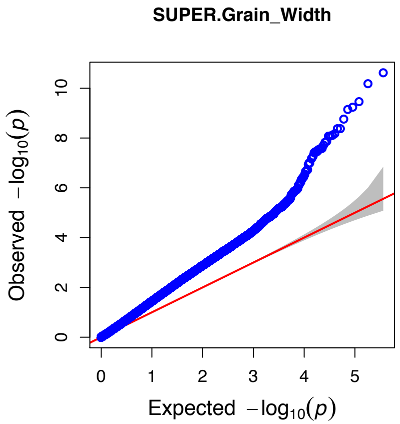

Indica Panel
426 samples
297 phenotypes

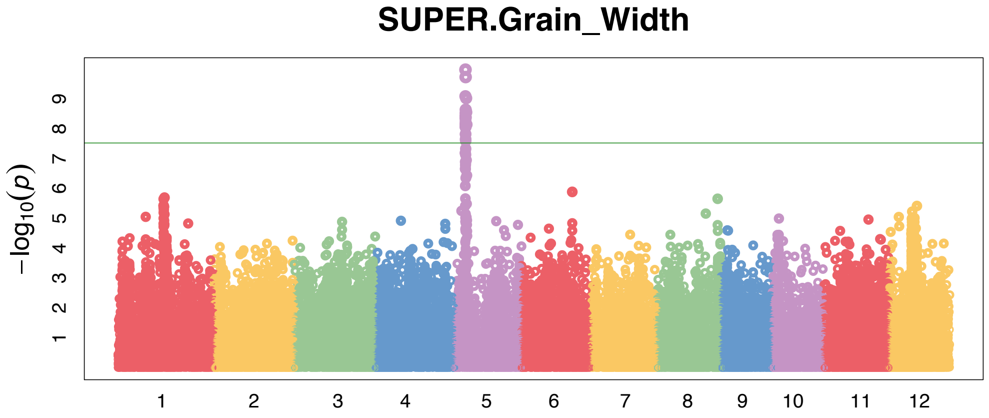

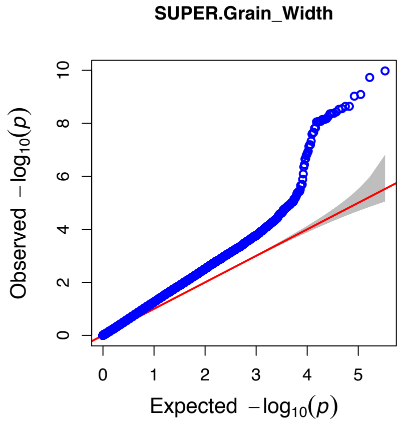

Japonica Panel
211 samples
178 phenotypes

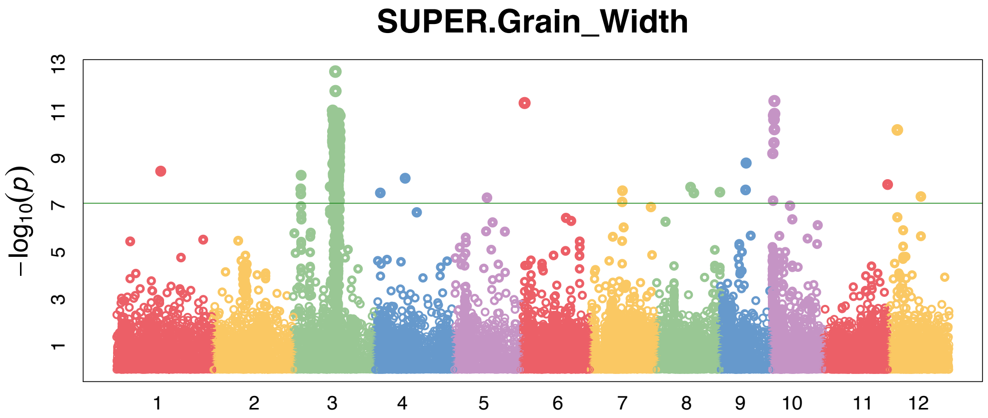

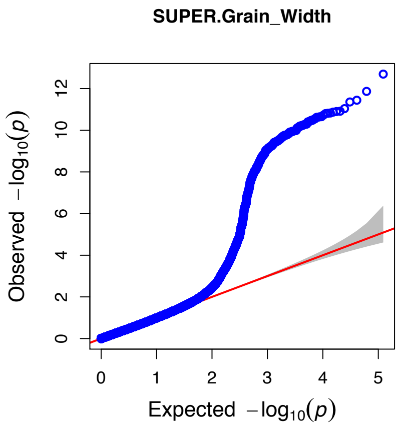

Full Panel
672 samples
500 phenotypes

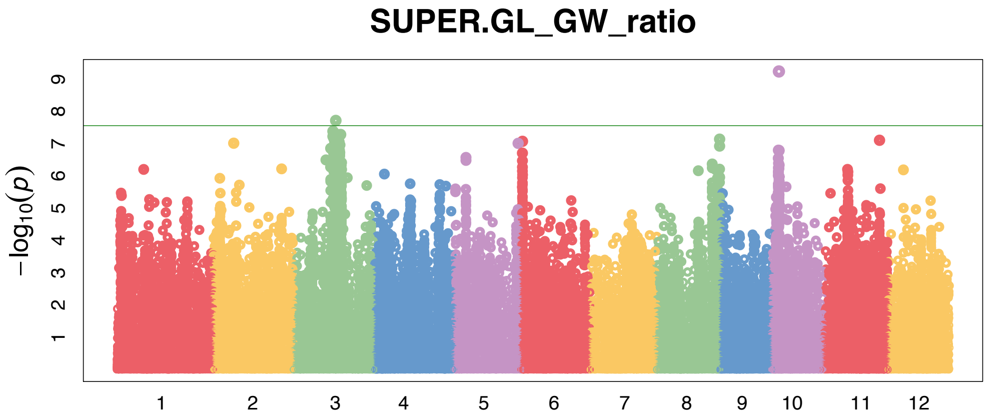

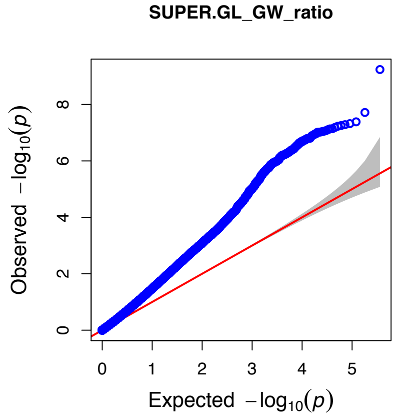

Indica Panel
426 samples
295 phenotypes

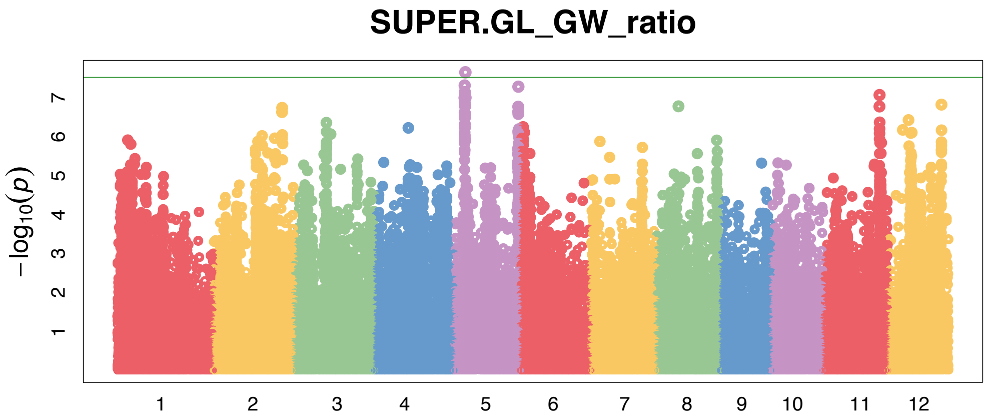

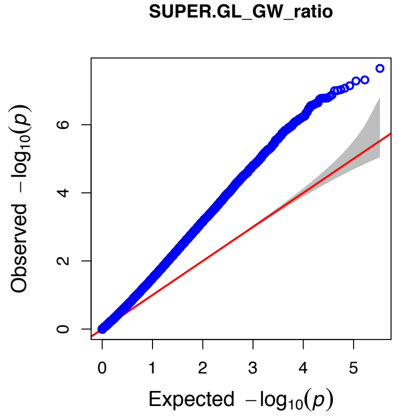

Japonica Panel
211 samples
177 phenotypes

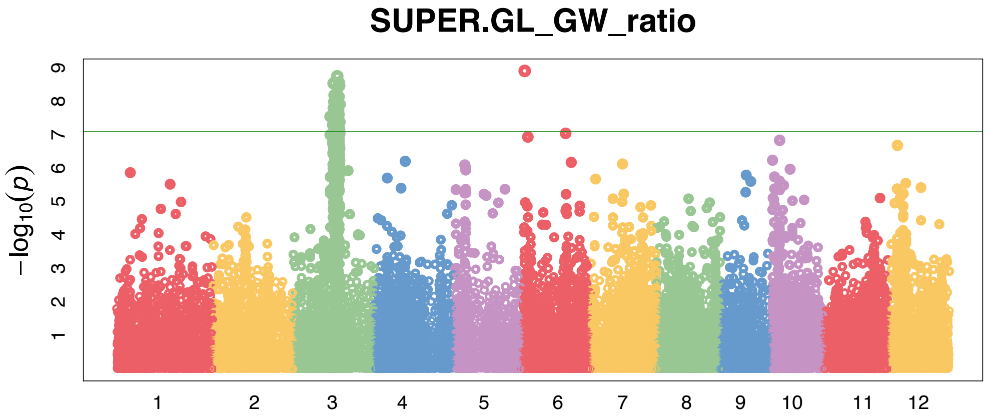

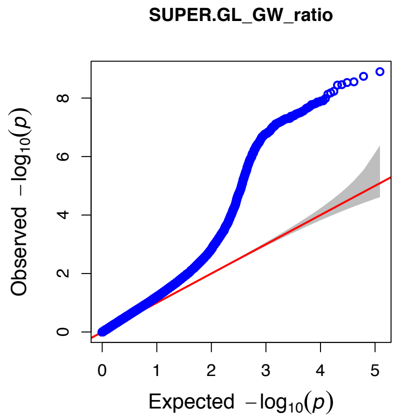

Full Panel
672 samples
486 phenotypes

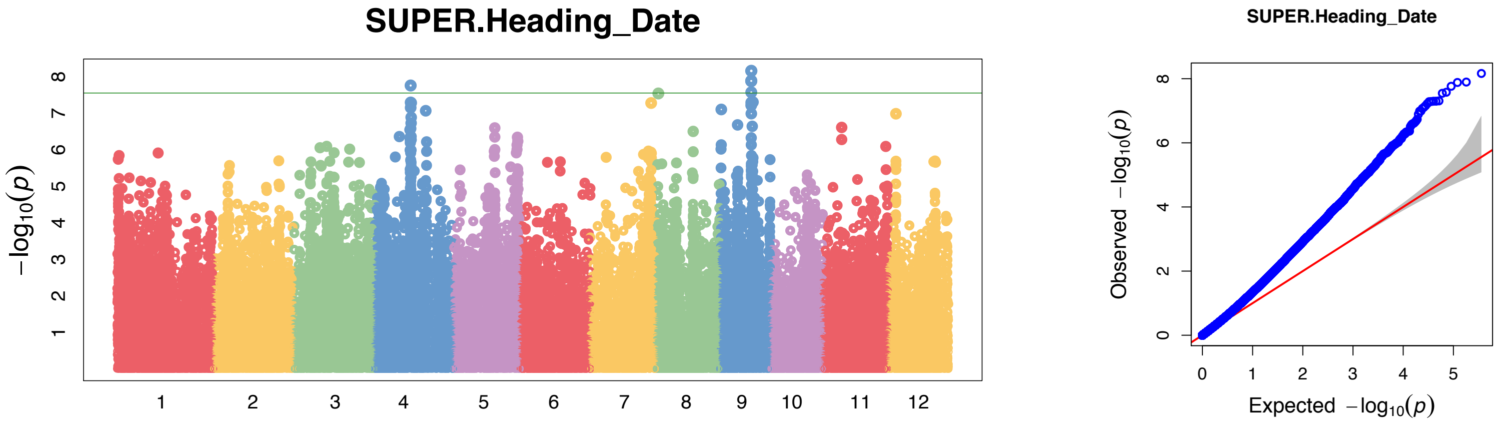

Indica Panel
426 samples
286 phenotypes

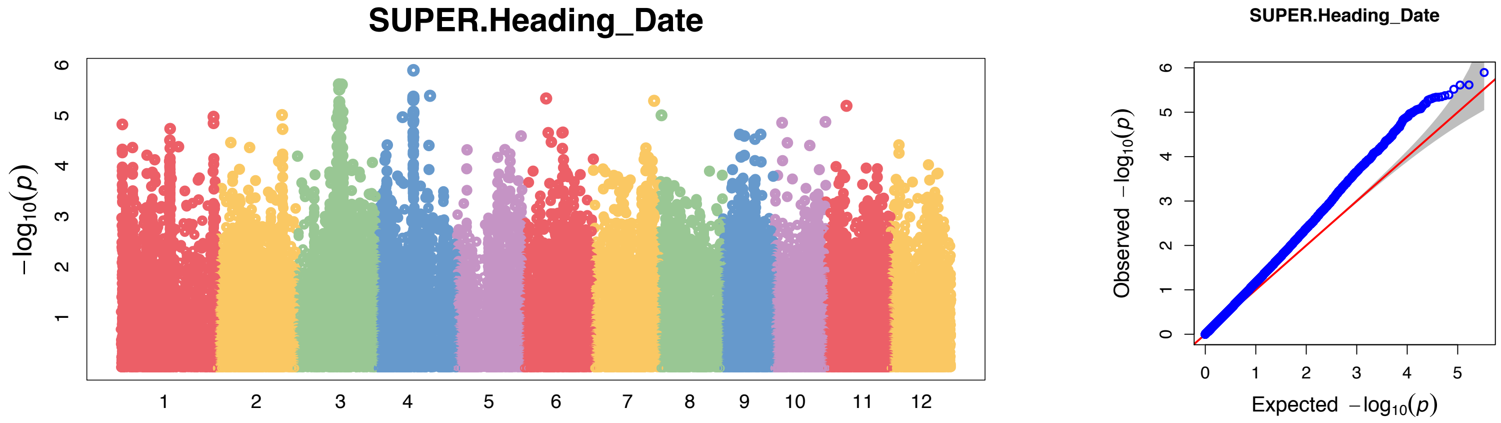

Japonica Panel
211 samples
172 phenotypes

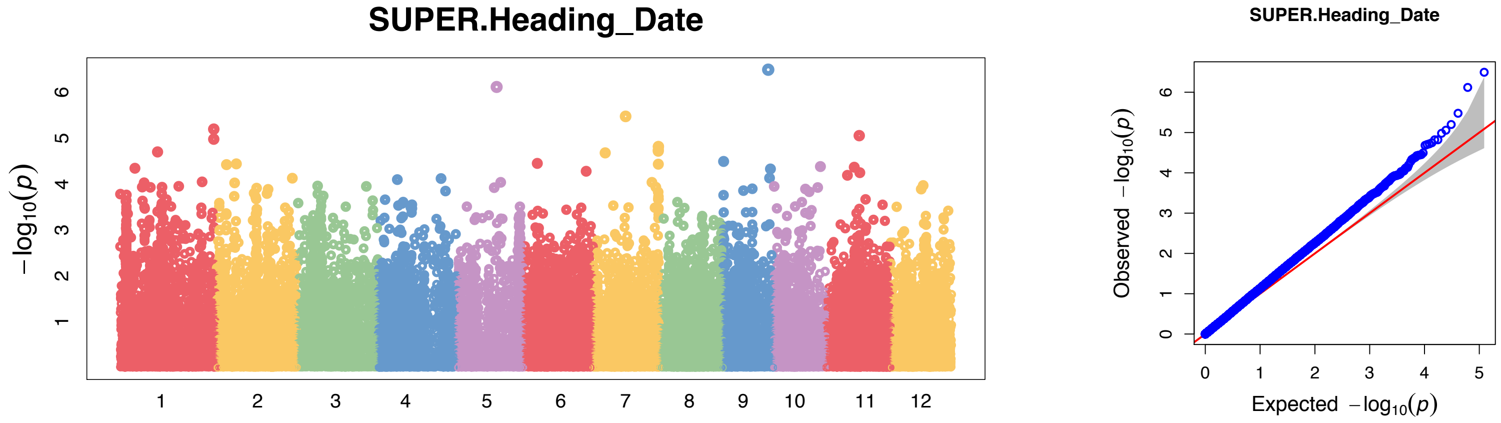

Full Panel
672 samples
452 phenotypes

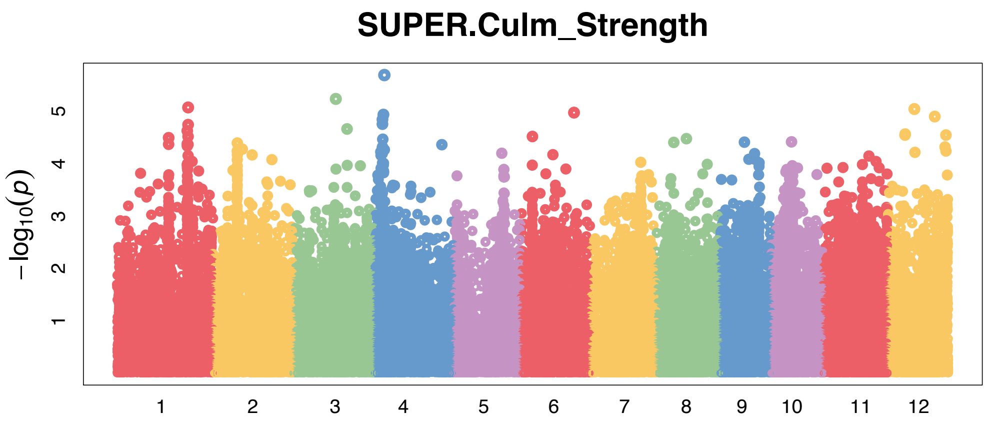

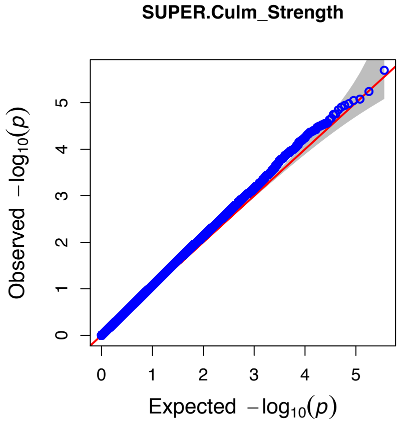

Indica Panel
426 samples
254 phenotypes

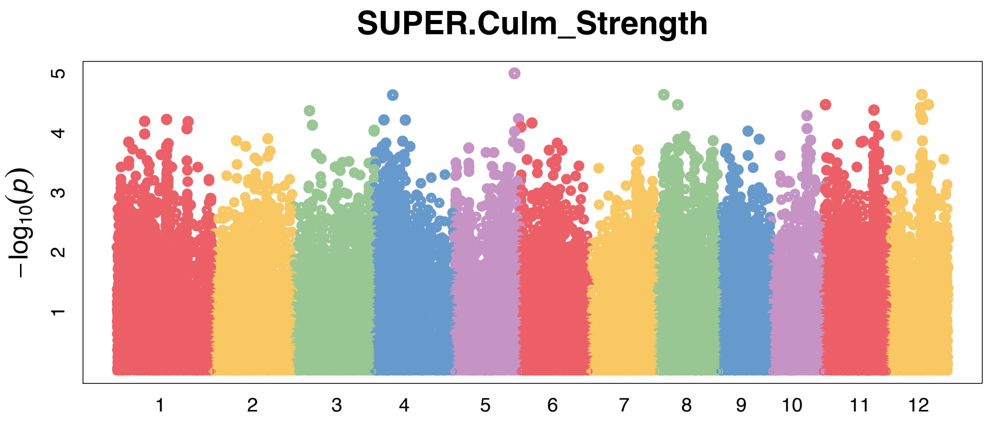

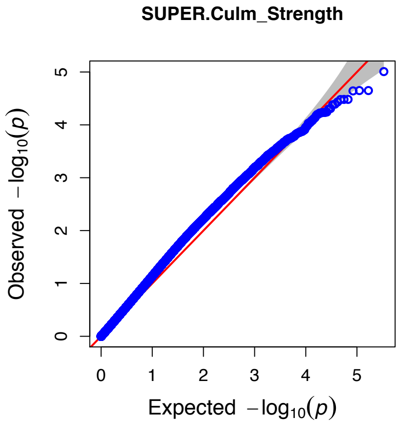

Japonica Panel
211 samples
170 phenotypes

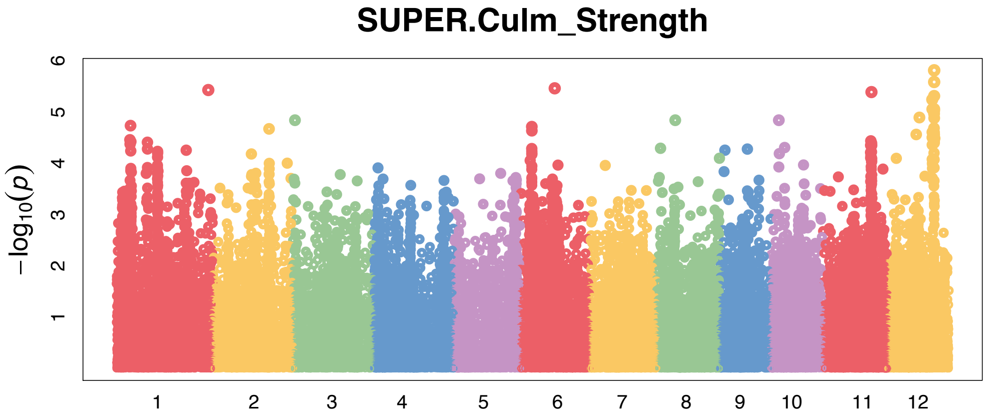

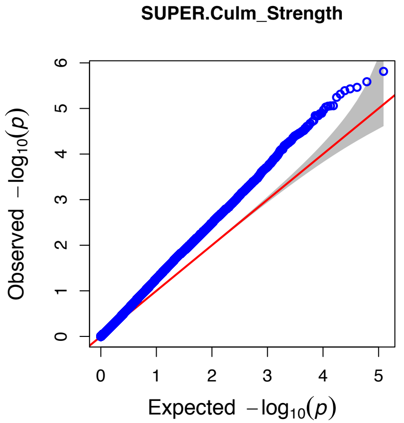

Full Panel
672 samples
356 phenotypes

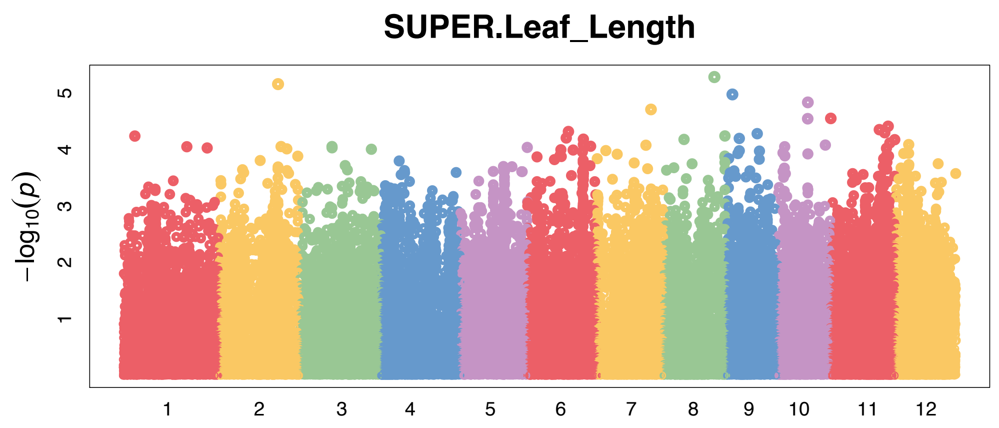

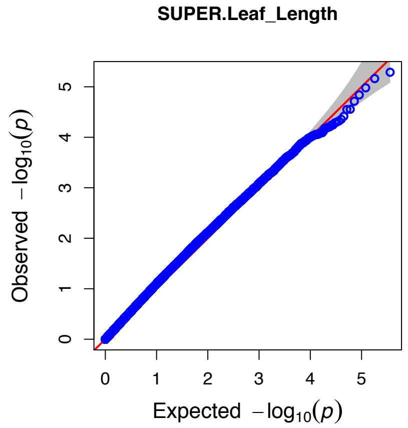

Indica Panel
426 samples
195 phenotypes

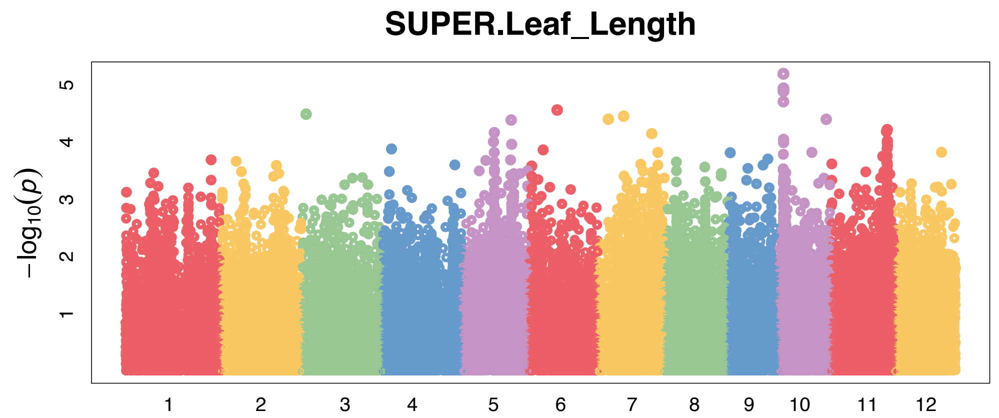

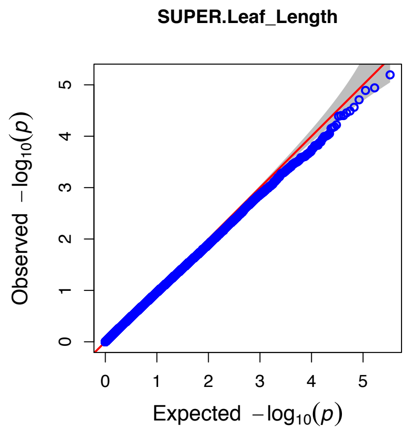

Japonica Panel
211 samples
137 phenotypes

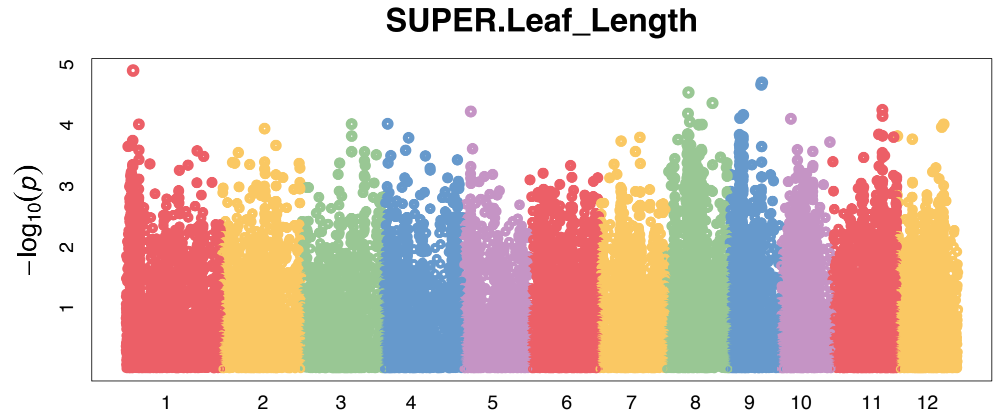

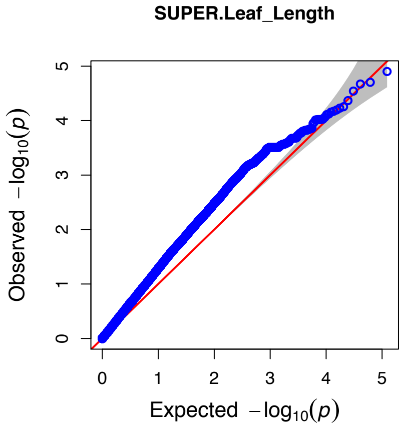

Full Panel
672 samples
355 phenotypes

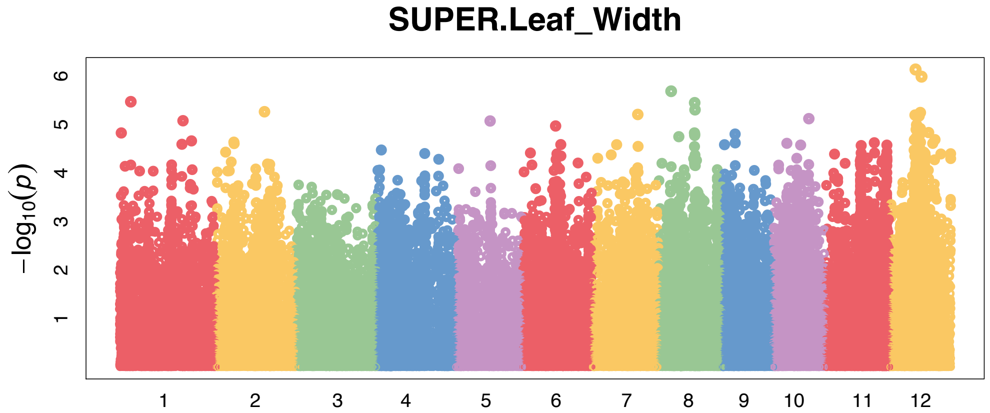

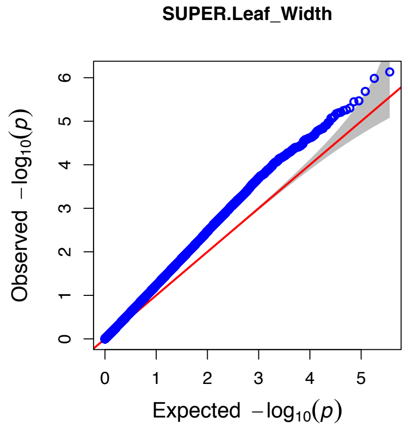

Indica Panel
426 samples
196 phenotypes

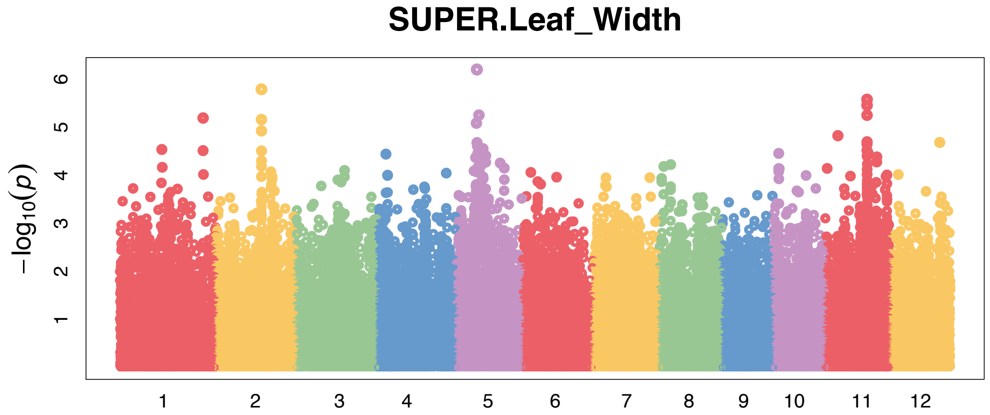

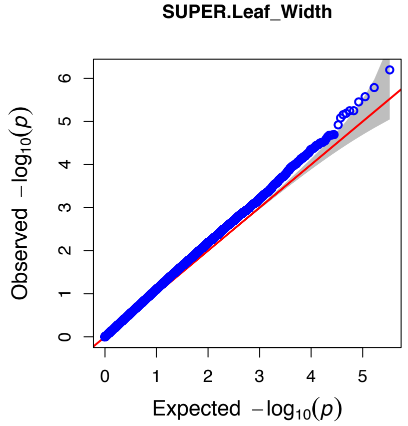

Japonica Panel
211 samples
136 phenotypes

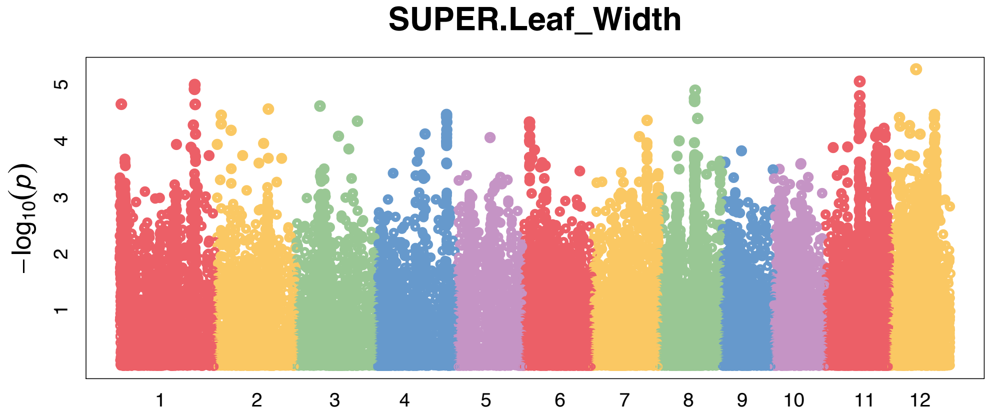

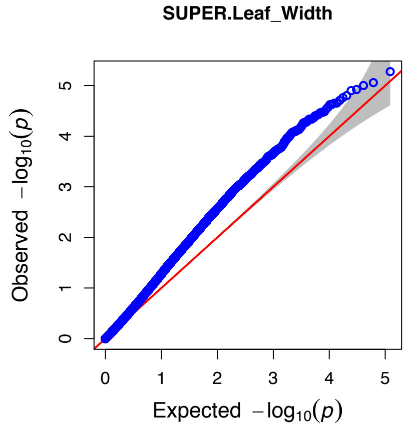

Supplement: Supplementary file 3 — Additional file 3: Figure S17. GWAS Manhattan and qq plots for the full panel and Indica and Japonica subpanels for Grain Length, Grain Width, Grain length-to-width ratio, Heading Date, Culm Strength, Leaf Length and Leaf Width. [file 12284_2021_481_MOESM3_ESM.pdf]
