# Supplementary material for: Resequencing of 672 Native Rice Accessions to Explore Genetic Diversity and Trait Associations in Vietnam
Source: Rice (N Y). 2021 Jun 10;14:52. doi: 10.1186/s12284-021-00481-0 (PMC8192651; doi:10.1186/s12284-021-00481-0)

Figure S18. GWAS Manhattan and qq plots for the full panel and Indica and Japonica subpanels for Leaf Pubescence, Culm Number, Diameter Internode, Culm Length, Panicle Length and Floret Pubescence

Full Panel
672 samples
328 phenotypes

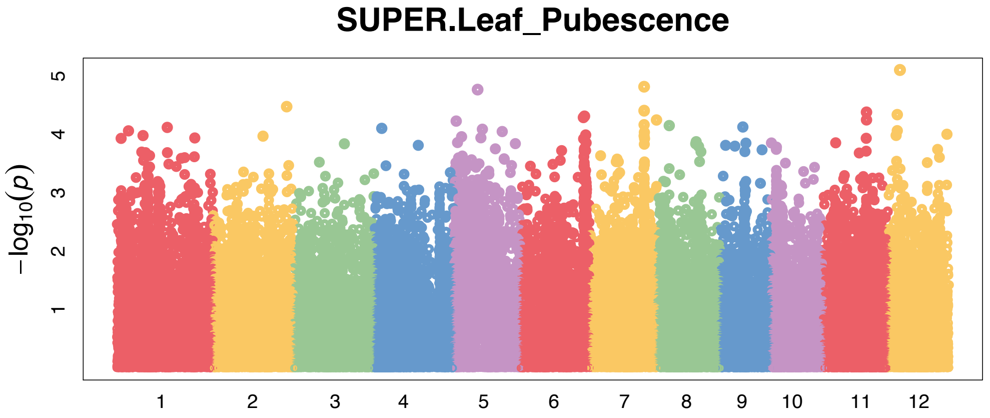

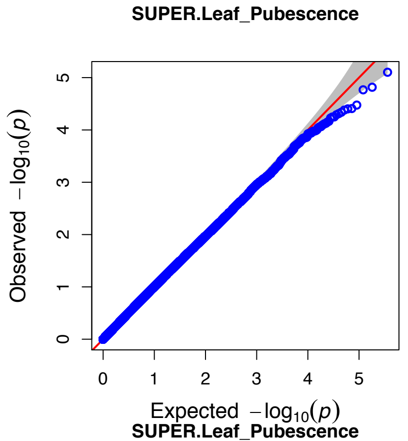

Indica Panel
426 samples
170 phenotypes

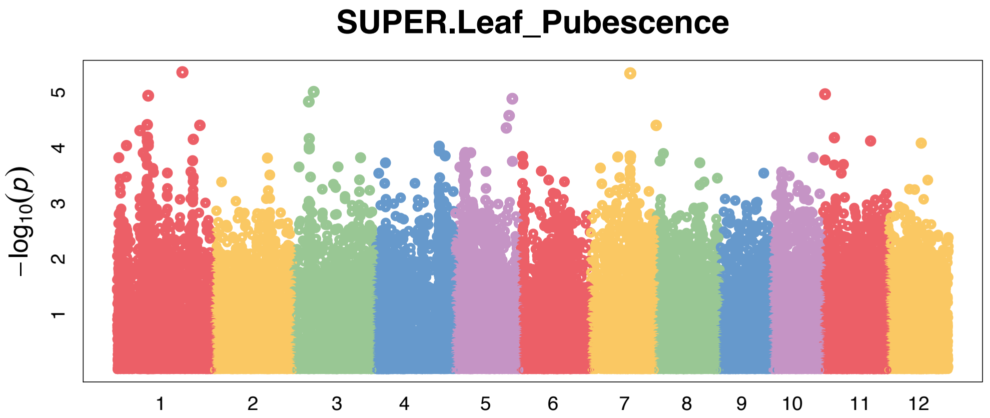

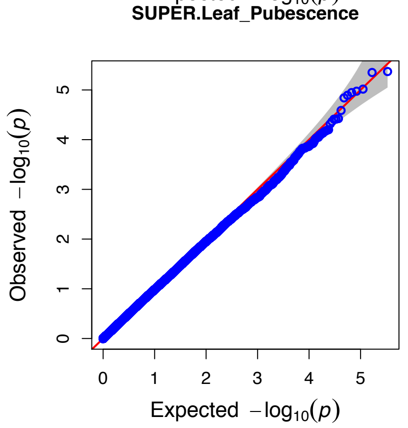

Japonica Panel
211 samples
134 phenotypes

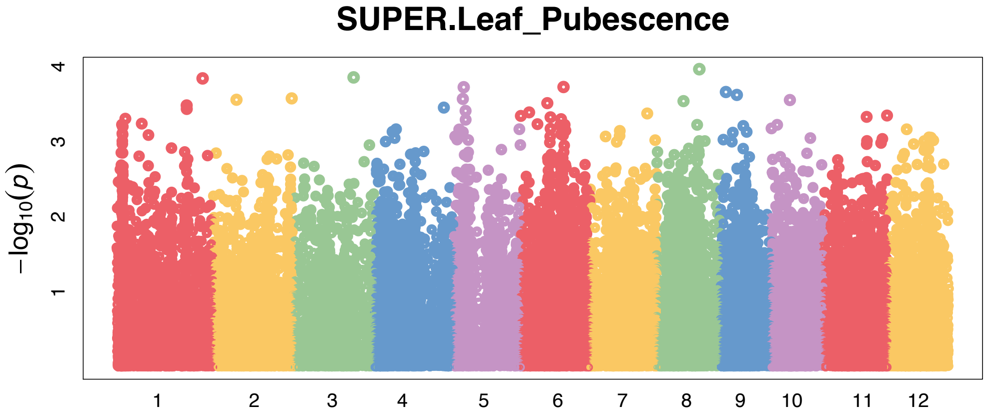

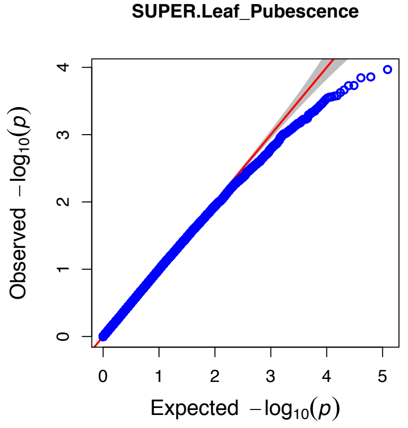

Full Panel
672 samples
454 phenotypes

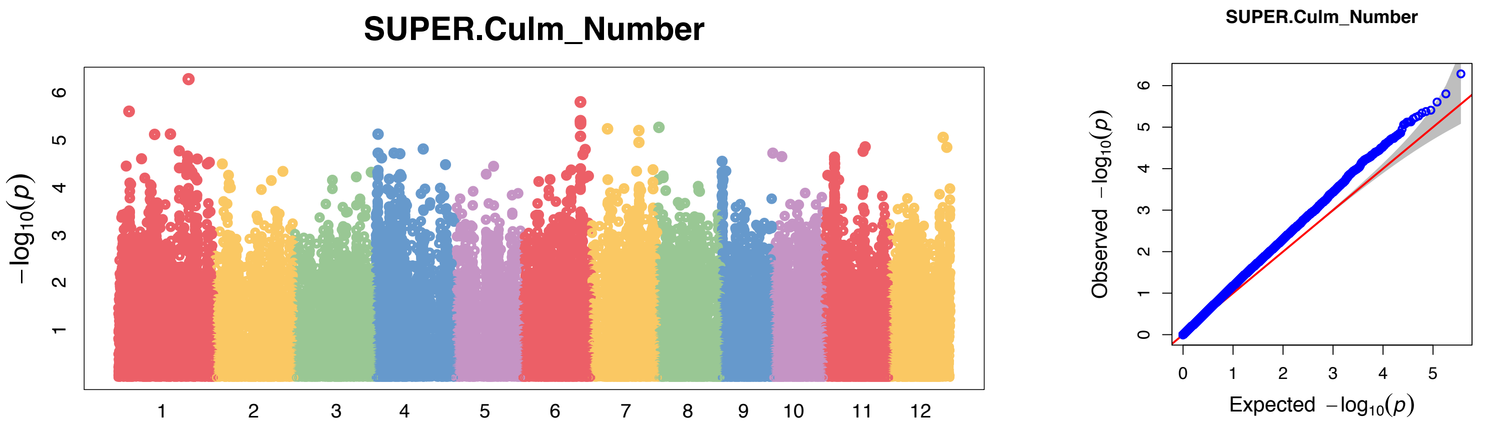

Indica Panel
426 samples
254 phenotypes

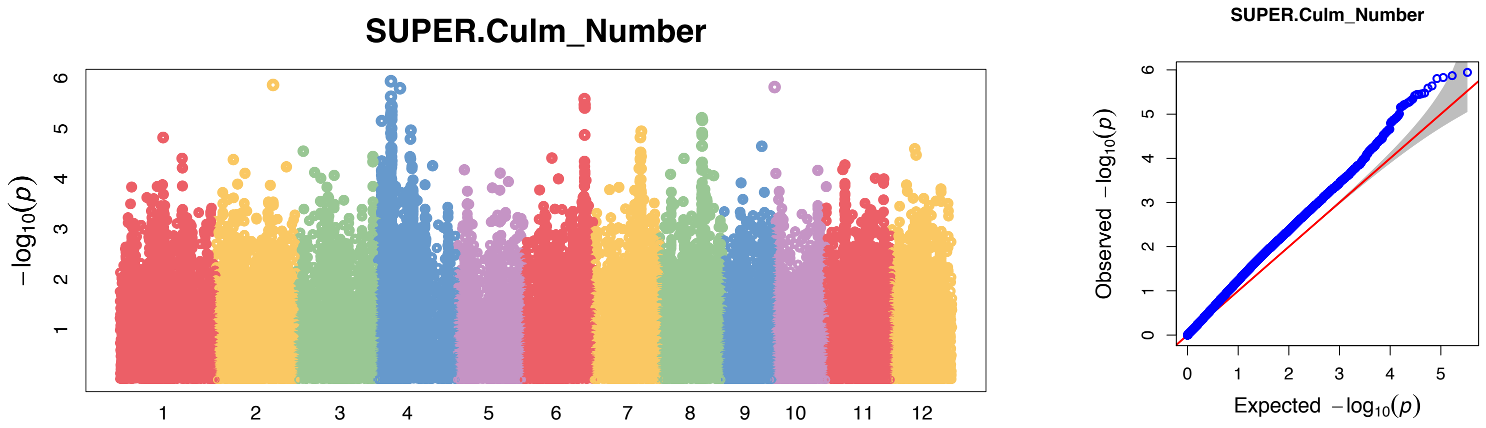

Japonica Panel
211 samples
172 phenotypes

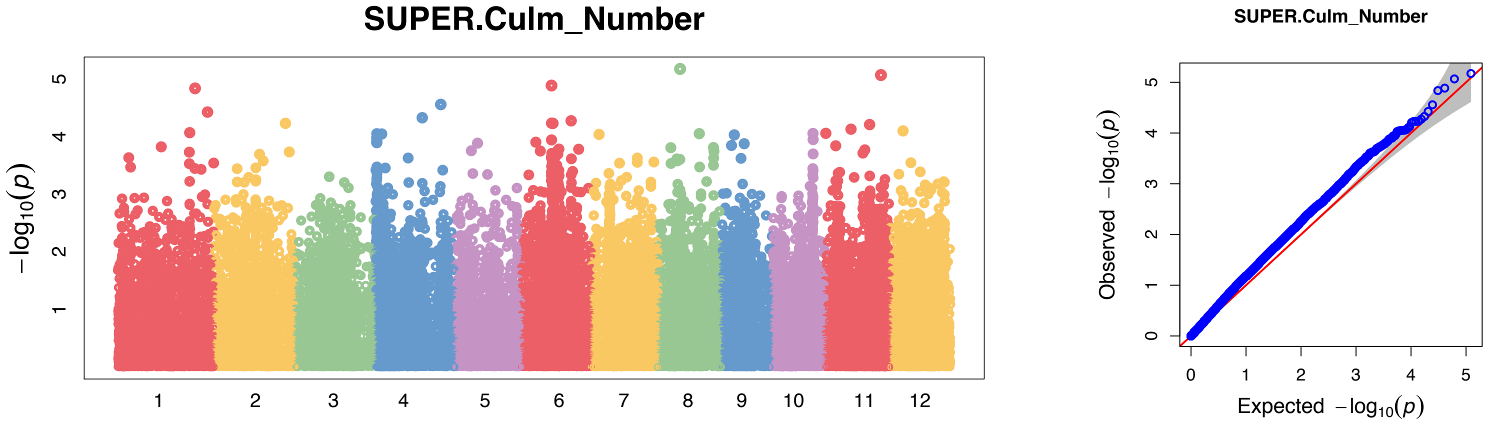

Full Panel
672 samples
485 phenotypes

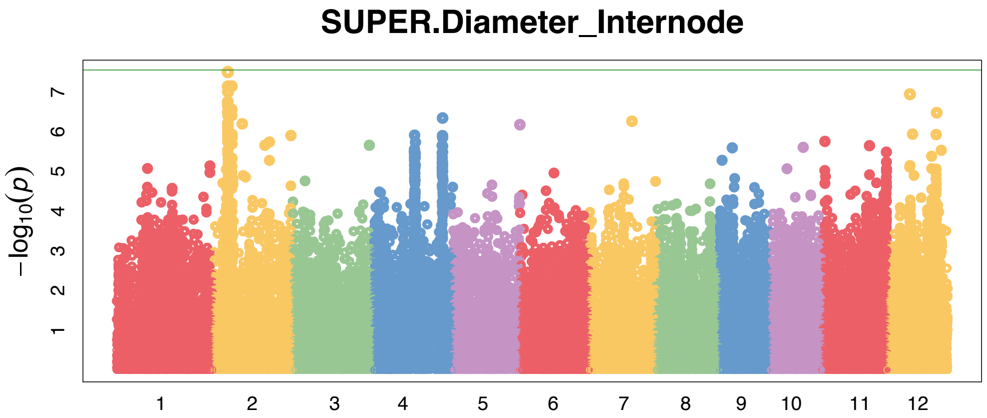

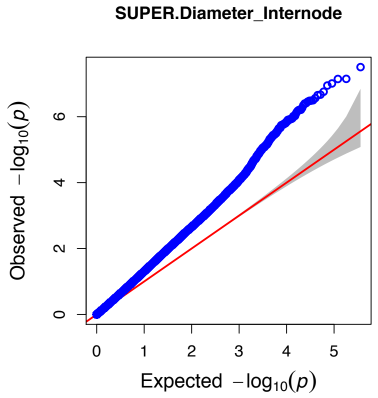

Indica Panel
426 samples
284 phenotypes

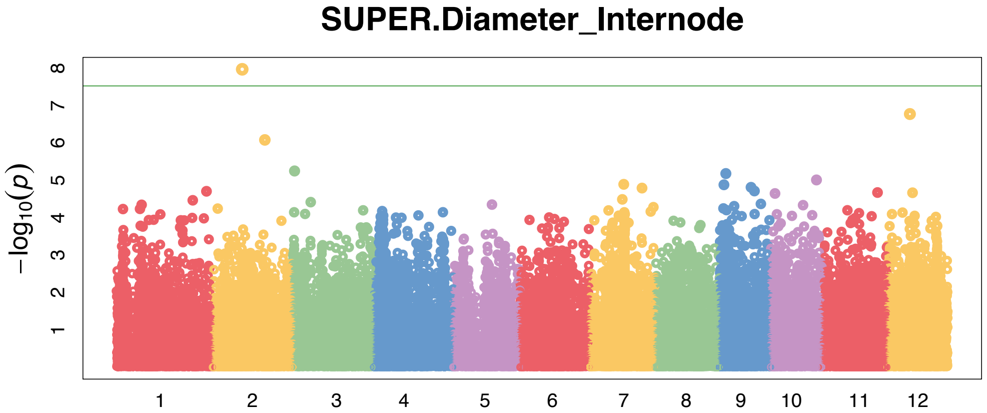

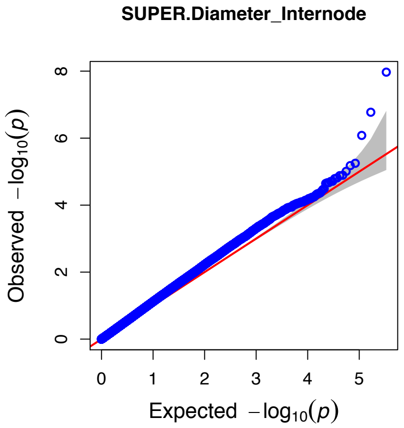

Japonica Panel
211 samples
174 phenotypes

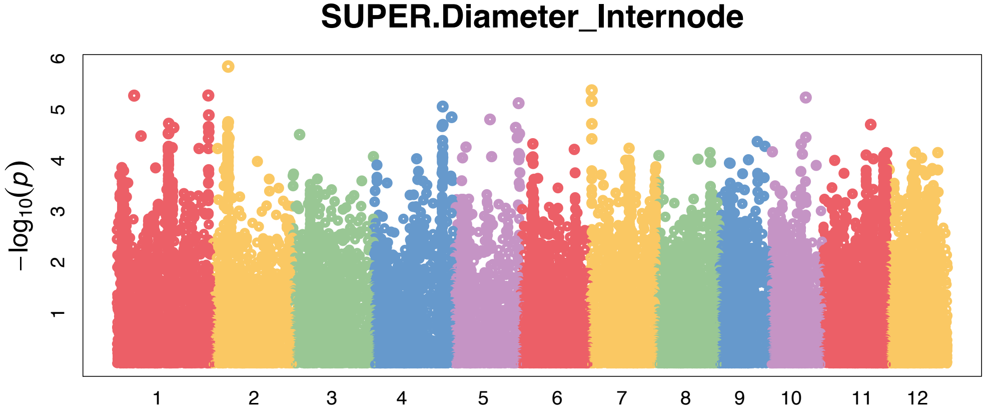

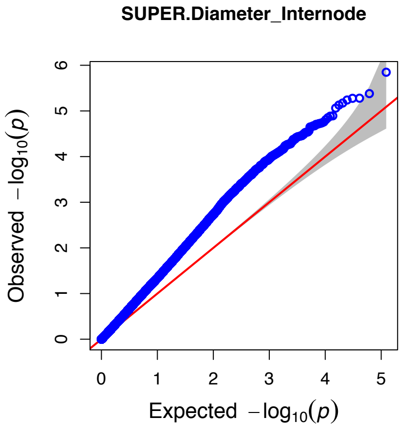

Full Panel
672 samples
485 phenotypes

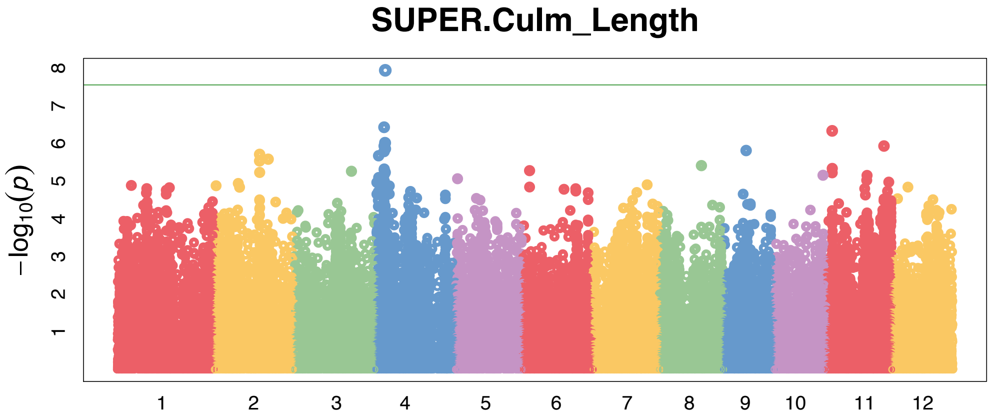

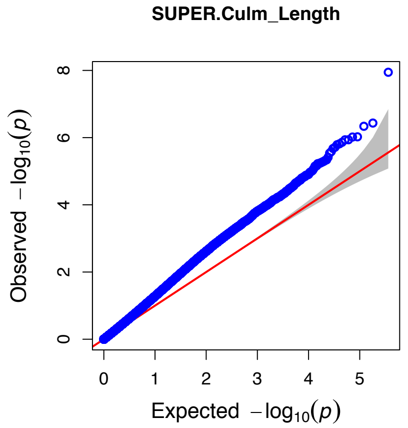

Indica Panel
426 samples
282 phenotypes

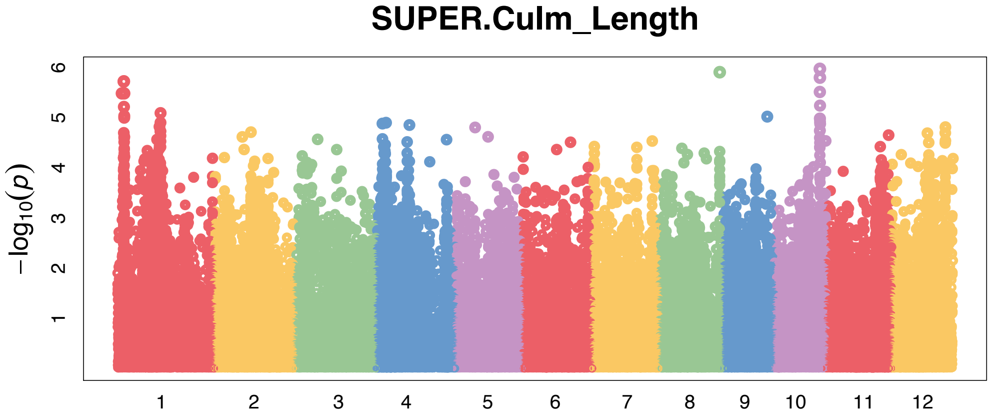

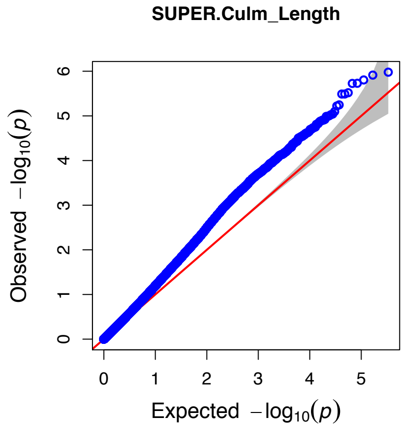

Japonica Panel
211 samples
175 phenotypes

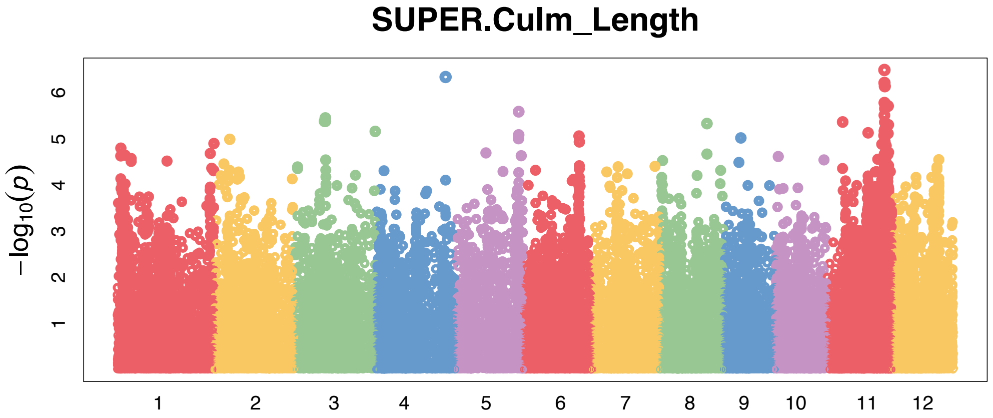

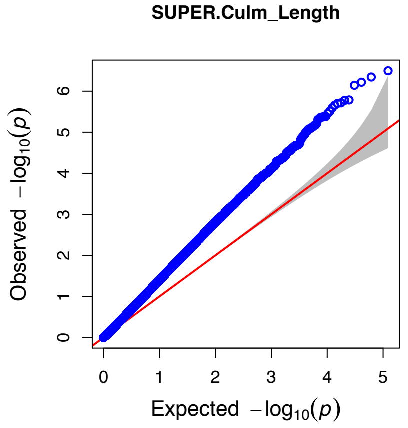

Full Panel
672 samples
486 phenotypes

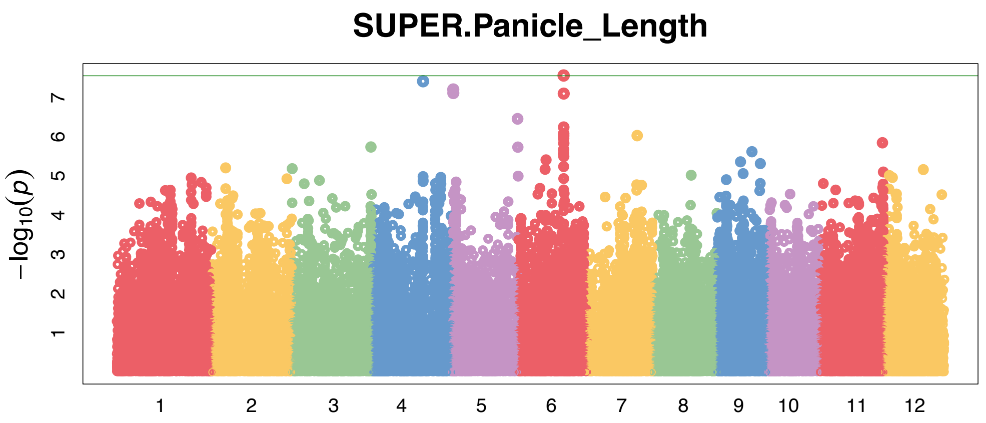

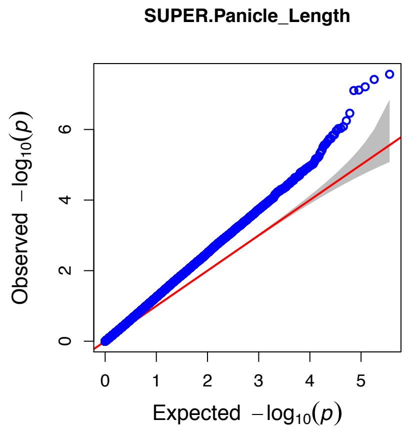

Indica Panel
426 samples
283 phenotypes

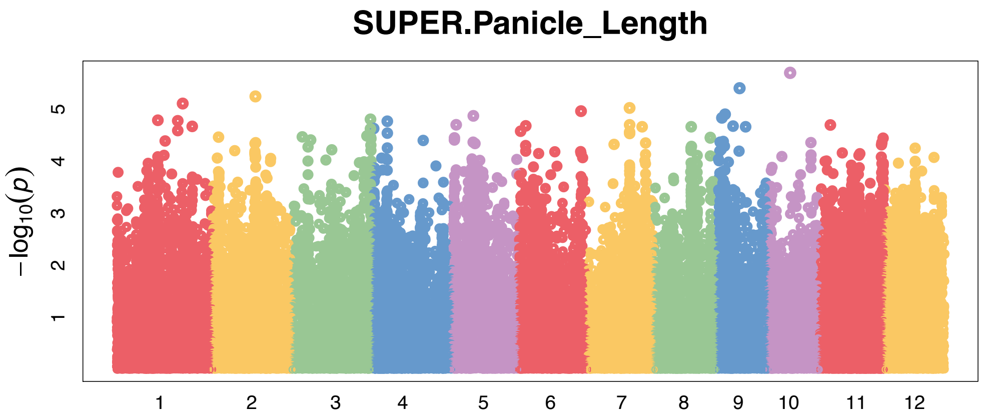

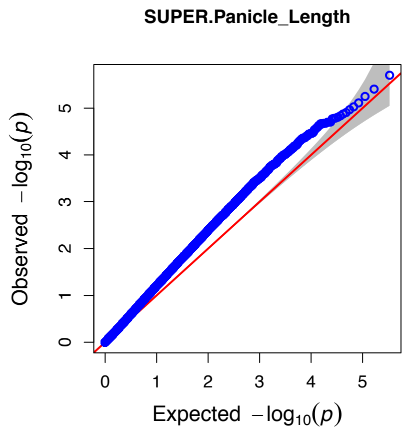

Japonica Panel
211 samples
175 phenotypes

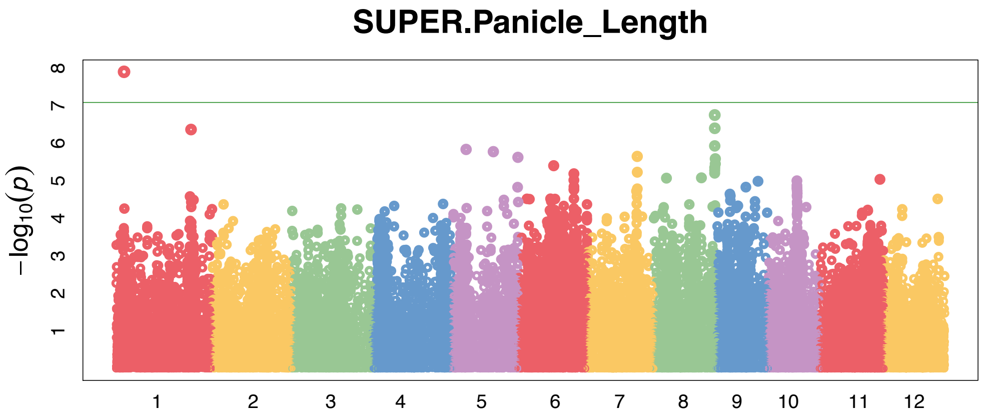

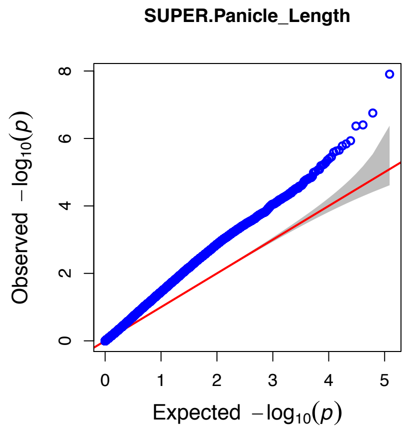

Full Panel
672 samples
488 phenotypes

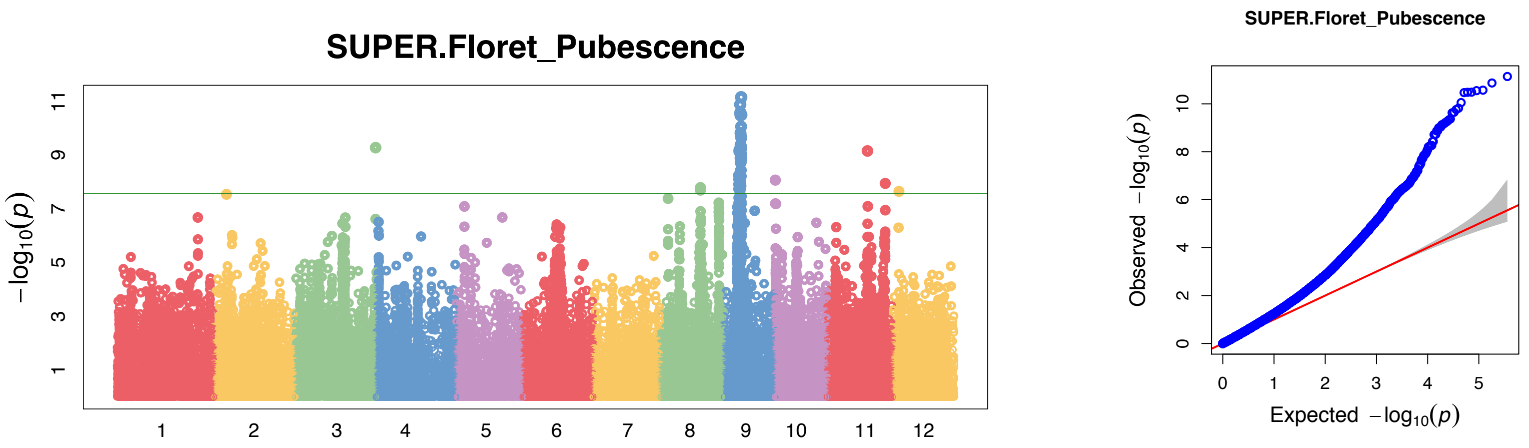

Indica Panel
426 samples
284 phenotypes

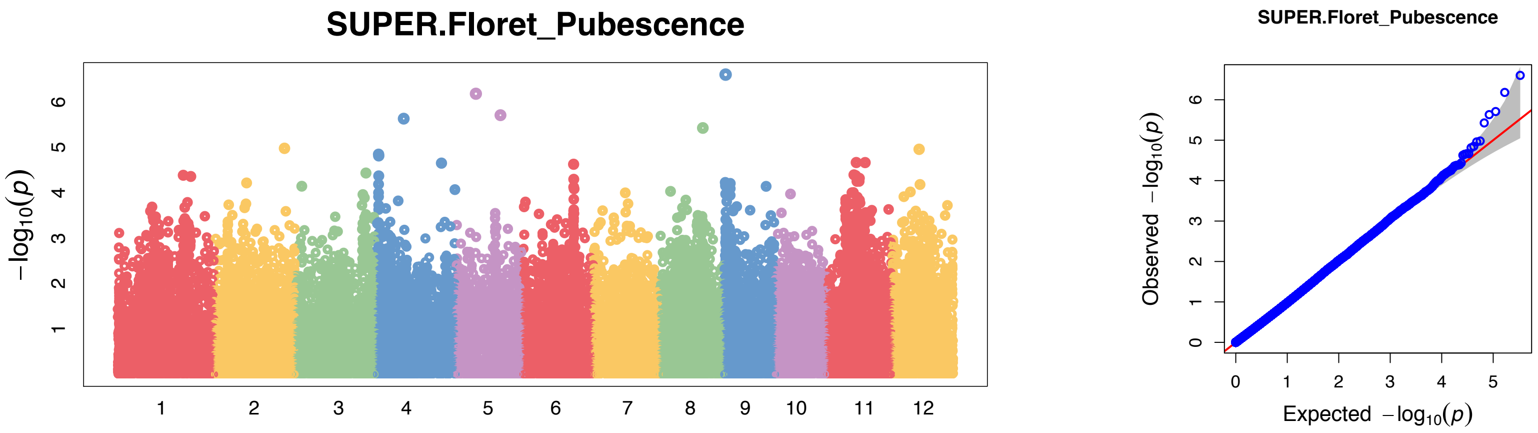

Japonica Panel
211 samples
176 phenotypes

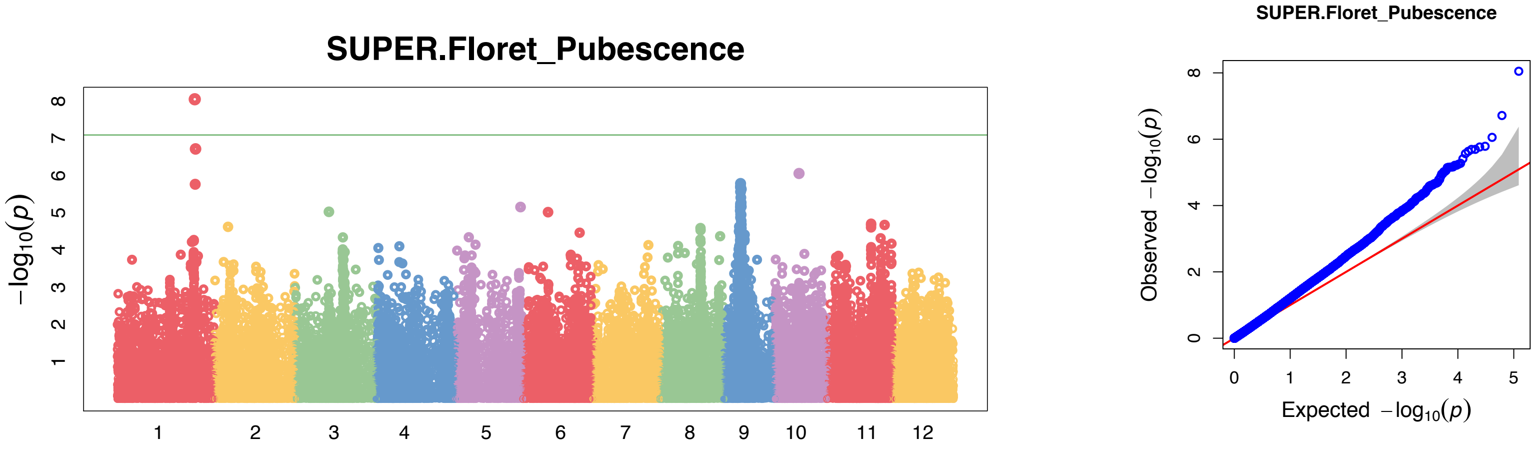

Supplement: Supplementary file 4 — Additional file 4: Figure S18. GWAS Manhattan and qq plots for the full panel and Indica and Japonica subpanels for Leaf Pubescence, Culm Number, Diameter Internode, Culm Length, Panicle Length and Floret Pubescence. [file 12284_2021_481_MOESM4_ESM.pdf]
